# Discovery of new myositis genetic associations through leveraging other immune-mediated diseases

**DOI:** 10.1016/j.xhgg.2024.100336

**Published:** 2024-07-22

**Authors:** Guillermo Reales, Christopher I. Amos, Olivier Benveniste, Hector Chinoy, Jan De Bleecker, Boel De Paepe, Andrea Doria, Peter K. Gregersen, Janine A. Lamb, Vidya Limaye, Ingrid E. Lundberg, Pedro M. Machado, Britta Maurer, Frederick W. Miller, Øyvind Molberg, Lauren M. Pachman, Leonid Padyukov, Timothy R. Radstake, Ann M. Reed, Lisa G. Rider, Simon Rothwell, Albert Selva-O'Callaghan, Jiri Vencovský, Lucy R. Wedderburn, Myositis Genetics Consortium, Chris Wallace

**Affiliations:** 1 Cambridge Institute of Therapeutic Immunology and Infectious Disease (CITIID), University of Cambridge, Cambridge, UK; 2 Department of Medicine, University of Cambridge, Cambridge, UK; 3 Department of Medicine, Baylor College of Medicine, Houston, TX, USA; 4 Department of Internal Medicine and Clinical Immunology, Pitié-Salpêtrière Hospital, Paris, France; 5 Department of Rheumatology, Salford Royal Hospital, Northern Care Alliance NHS Foundation Trust, Manchester Academic Health Science Centre, Salford, UK; 6 Division of Musculoskeletal and Dermatological Sciences, Faculty of Biology, Medicine and Health, The University of Manchester, Manchester, UK; 7 Department of Neurology, Ghent University, Ghent, Belgium; 8 Neuromuscular Reference Center, Ghent University Hospital, Ghent, Belgium; 9 Rheumatology Unit, Department of Medicine, University of Padova, Padova, Italy; 10 The Robert S. Boas Center for Genomics and Human Genetics, The Feinstein Institute, Manhasset, NY, USA; 11 Epidemiology and Public Health Group, Division of Population Health, Health Services Research & Primary Care, Faculty of Biology, Medicine and Health, University of Manchester, Manchester, UK; 12 Rheumatology Unit, Royal Adelaide Hospital, Adelaide, South Australia, Australia; 13 Discipline of Medicine, Adelaide University, Adelaide, South Australia, Australia; 14 Division of Rheumatology, Department of Medicine, Solna, Karolinska Institutet, Karolinska University Hospital, Stockholm, Sweden; 15 Department of Neuromuscular Diseases, UCL Queen Square Institute of Neurology. London, UK; 16 Centre for Rheumatology, UCL Division of Medicine, University College London, London, UK; 17 Department of Rheumatology and Immunology, Inselspital, Bern University Hospital, University of Bern, Bern, Switzerland; 18 Environmental Autoimmunity Group, National Institute of Environmental Health Sciences, NIH, Bethesda, MD, USA; 19 Department of Rheumatology, Oslo University Hospital, Oslo, Norway; 20 Children’s Hospital of Chicago, Northwestern University Feinberg School of Medicine, Chicago, IL, USA; 21 Department of Rheumatology and Clinical Immunology, University Medical Center, Utrecht, the Netherlands; 22 Department of Pediatrics, Duke University, Durham, NC, USA; 23 Centre for Genetics and Genomics Versus Arthritis, Centre for Musculoskeletal Research, Faculty of Biology, Medicine and Health, University of Manchester, Manchester, UK; 24 Internal Medicine Department, Vall d'Hebron General Hospital, Universitat Autonoma de Barcelona, Barcelona, Spain; 25 Institute of Rheumatology and Department of Rheumatology, First Medical Faculty, Charles University, Prague, Czech Republic; 26 Centre for Adolescent Rheumatology Versus Arthritis, UCL Great Ormond Street Institute of Child Health, University College London, London, UK; 27 NIHR Biomedical Research Centre at Great Ormond Street Hospital, London, UK; 28 MRC Biostatistics Unit, University of Cambridge, Cambridge, UK

## Abstract

Genome-wide association studies (GWASs) have been successful at finding associations between genetic variants and human traits, including the immune-mediated diseases (IMDs). However, the requirement of large sample sizes for discovery poses a challenge for learning about less common diseases, where increasing volunteer numbers might not be feasible. An example of this is myositis (or idiopathic inflammatory myopathies [IIM]s), a group of rare, heterogeneous autoimmune diseases affecting skeletal muscle and other organs, severely impairing life quality. Here, we applied a feature engineering method to borrow information from larger IMD GWASs to find new genetic associations with IIM and its subgroups. Combining this approach with two clustering methods, we found 17 IMDs genetically close to IIM, including some common comorbid conditions, such as systemic sclerosis and Sjögren’s syndrome, as well as hypo- and hyperthyroidism. All IIM subtypes were genetically similar within this framework. Next, we colocalized IIM signals that overlapped IMD signals, and found seven potentially novel myositis associations mapped to immune-related genes, including *BLK*, *IRF5/TNPO3*, and *ITK*/*HAVCR2*, implicating a role for both B and T cells in IIM. This work proposes a new paradigm of genetic discovery in rarer diseases by leveraging information from more common IMD, and can be expanded to other conditions and traits beyond IMD.

## Teaser

Finding relevant genetic variants is challenging for rare diseases due to decreased statistical power. We used a feature engineering approach that borrows information from common IMDs, followed by colocalization, to find seven additional associations in myositis, implicating a role for both T cells and B cells.

## Introduction

Since their first application more than 15 years ago, genome-wide association studies (GWASs) have successfully identified tens of thousands of genetic variants associated with thousands of diseases and biological traits. These efforts have provided key insights into trait genetic architecture and helped achieve translational benefits, including creating polygenic risk scores and drug repurposing.^1^^,^^2^ One of the crucial challenges of GWAS has been statistical power, which is crucial for discovery. Since millions of variants are tested in GWASs, the threshold to consider a variant statistically significant must be high to avoid type 1 errors (i.e., false positives), so large cohorts are needed. While cohort sizes have been increasing over time for a wide variety of diseases and traits, recruiting enough individuals in the context of rare diseases is often not possible due to the low numbers of patients for such diseases. Here, we leverage the genetic evidence across a wide range of common immune-mediated diseases (IMD) to enhance discovery in one group of rare diseases: idiopathic inflammatory myopathies (IIMs [MIM: 160750]).

IIMs, also known as myositis, are a heterogeneous group of rare, systemic autoimmune diseases affecting skeletal muscle, skin, and other organs, and characterized by chronic inflammation and muscle weakness, leading to severe impairment of quality of life.^3^^,^^4^ IIM patients can be classified in subtypes based on clinical and serological criteria, although IIM rarity and heterogeneity have limited our understanding of IIM pathogenesis and thus made diagnosis and classification efforts challenging.^5^^,^^6^ There are some recognized subgroups of IIM, including, but not limited to, juvenile-onset dermatomyositis (JDM), dermatomyositis (DM), inclusion body myositis (IBM), and polymyositis (PM). Other subgroups are determined by the detection of autoantibodies, such as the anti-histidyl-tRNA synthetase (anti-Jo1), associated with the anti-synthetase syndrome.^6^

GWASs conducted on IIM in individuals with European ancestry have identified the strongest disease associations at the major histocompatibility cluster locus (MHC, chromosome 6),^7^^,^^8^^,^^9^ a region commonly associated with IMD, with additional associations at *PTPN22* (associated with PM)^9^^,^^10^^,^^11^ *STAT4*, *TRAF6*, *UBE2L*,^9^^,^^10^^,^^12^ *PLCL1*, *BLK*, and *CCR5*.^9^^,^^13^ IIM-associated genes are involved in crucial innate and adaptive immune response pathways, such as the T cell receptor pathway (i.e., *PTPN22* and *STAT4*), and the nuclear factor-κB (NF-κB) signaling pathway (*BLK*, *UBE2L3*, and *TRAF6*).^5^ Other efforts have explored the contribution of rare genetic variation, finding an association with *IFI35* via aggregate association testing.^14^

The number of confirmed genetic associations for IIM is relatively small compared to other, more common IMD (e.g., asthma or type 1 diabetes) due to limited GWAS sample sizes, resulting in limited statistical power for discovery, particularly within IIM subtypes. However, the coexistence of IIM with other IMD, such as systemic lupus erythematosus (SLE [MIM: 152700]), Sjögren’s syndrome (SjS [MIM: 270150]), and systemic sclerosis (SSc [MIM: 181750]) in IIM patients, as well as familial coaggregation, suggest that IIM genetic risk factors may be shared with other IMDs.^15^^,^^16^^,^^17^^,^^18^^,^^19^

We recently developed a method to learn shared genetic risk factors among related diseases and enable the transfer of learning from larger IMD GWASs to inform smaller studies.^20^ With this approach, we derived a set of 13 features that capture different aspects of IMD risk and can be used together to study a new independent dataset or to compare different datasets in a much lower dimensional, IMD-focused space. While some features have straightforward interpretations (e.g., PC1 discriminates between auto-inflammatory and autoimmune disease), others do not, and the interpretation of association of any given dataset to a given feature requires care. Each feature is defined as a weighted sum of effect sizes across a subset of “driver” SNPs, where the weight, and the choice of SNPs (which varies between features), was learnt from the set of large IMD GWAS. Thus, independent datasets can be projected onto these features and each feature, and associated driver SNPs, tested for significant association with the disease of interest (described in detail in materials and methods). We used this strategy to identify novel associations for small GWASs that, when replicated in larger datasets when these were available, gave us confidence that this approach can be used to identify association in GWASs of IMD with relatively small sample sizes.

Here we analyzed summary data from the two largest IIM genetics studies to date in the context of the 13 IMD features learned this way: (1) a full GWAS of 1,711 cases and 4,724 controls (which we call the “Miller study”)^8^ and (2) a more extensive study of 2,565 cases and 10,260 controls using the immune-targeted ImmunoChip and subsequently imputed to genome-wide coverage (the “Rothwell study”).^9^ The samples in the two studies substantially overlap, but the genotyping platforms and genome coverage are substantially different. These two studies comprised multiple GWAS IIM subgroups (DM, JDM, and PM in Miller, and DM, JDM, PM, anti-Jo1^+^, and IBM in Rothwell), as well as one meta-analysis containing all subgroups (which we refer to as “IIM (M)/(R)” in our figures and tables) in each study, totaling 10 datasets (see Table S1). Given that these studies balance strengths in sample size (larger in Rothwell) vs. SNP coverage (larger in Miller), we chose to analyze both, concentrating on features that showed significant association with either one, as long as they showed consistent direction of association with the feature.

We projected the IIM and a selection of 466 IMD GWAS summary statistics onto our learned feature space and used this low-dimensional representation of IIM genetics to better understand the genetic basis of IIM and its clinical subtypes, identifying which IMD exhibited close genetic proximity to IIM overall and which shared specific associations with IIM or its subtypes.

## Materials and methods

### IIM GWAS data

We used GWAS summary statistics from two IIM studies, which we refer to as the Miller and the Rothwell studies (Table S1).^8^^,^^9^ Both studies included European individuals recruited by the Myositis Genetics Consortium,^10^ with some technical differences. More specifically, Miller included 1,710 cases and 4,724 controls that were genotyped using multiple Illumina genome-wide arrays, described elsewhere.^7^ Rothwell included an expanded sample, comprising 2,565 cases and 10,260 controls, and included approximately 1.6 million SNPs genotyped using the ImmunoChip array followed by imputation using the Haplotype Reference Consortium panel. Both studies classified patients in IIM subtypes and performed GWASs on each subtype and a pooled IIM sample (referred to as IIM (M) and IIM (R) in our tables and figures). Rothwell subtypes comprised Anti-Jo1^+^ myositis, DM, IBM, JDM, and PM. Miller included DM, JDM, and PM subtypes.

### Harmonization, imputation, and projection of GWAS data

To analyze IIM datasets in the context of other IMD, we created a compendium of IMD GWAS summary statistics datasets from public repositories, including the NHGRI-EBI GWAS catalog,^21^ FinnGen Project Release 7,^22^ UK Biobank (Pan-UK Biobank),^23^ and Biobank Japan,^24^ or via a request to study authors (Table S2). GWAS summary statistics datasets come in different formats, with no current consensus format. We wrote a shell and R pipeline (GWAS_tools) for formatting and quality control of all downloaded datasets, trying to accommodate as many different input formats as possible and keeping a consensus minimum set of information from each study: genomic coordinates, reference and effect allele, effect size estimate measured as log odds ratio (Beta), standard error of the effect size, and *p* value. We identified the genome build of the datasets and computed the genomic coordinates in the GRCh38/hg38 genome build using the liftOver tool (available at UCSC Genome browser website).

We also re-scaled the effect sizes and standard errors of datasets when generated using linear models (e.g., BOLT-LMM) from linear to odds ratio scale by using the proportion of cases, as suggested in BOLT-LMM manual^25^:

$$cp=N_{1}/\left(N_{0}+N_{1}\right)$$

$$\hat{\beta }=\beta /\left(cp\;\left(1-cp\right)\right)$$

$$\hat{SE}=\frac{SE}{\left(cp\;\left(1-cp\right)\right)}\text{,}$$

where β and SE are the original estimated effect sizes and standard error in the linear scale, $\hat{\beta }$ and $\hat{SE}$ the effect sizes and standard error in odds ratio scale, $N_{1}$ is the number of cases and, $N_{0}$ the number of controls. Finally, we excluded all datasets that contained less than 80% of the driver SNPs in the 13 IMD features, as well as datasets that were components of meta-analyses used to train the features, as these would be overfitted.^20^

We projected quality-controlled datasets onto the 13 features using the cupcake package. Each feature is defined by a set of driver SNPs (ranging from 107 to 373), and associated weights learnt from the large IMD GWAS studies. To learn the weights, we needed to first center a matrix of scaled effect sizes from the large IMD studies. New datasets can be projected into the feature space by first subtracting the same centering factor, then summing the weighted effect estimates across these driver SNPs for each feature. We report projected results as $\hat{\delta }$, the difference between the projected $\hat{\beta }$ and a projected synthetic control with all zero entries (i.e., all $\hat{\beta }$ set to zero). This allows us to perform statistical inference on the estimand, δ, being significantly different from zero.

We used the Benjamini-Hochberg approach, calling *overall* significance of a test of δ = 0 at a false discovery rate (FDR) of less than 0.01. To estimate the FDR, we consider the overall test statistic not just for the projected IIM datasets, but also all 466 datasets we projected for comparison (Tables S2 and S3). The test for overall significance is a 13 degree of freedom chi-squared test (for 13 features), and we must account for any correlation between features that arises due to linkage disequilibrium (LD) between driver SNPs associated with the different features. Calculation of this covariance matrix and its use in a chi-squared test of δ = 0 is described in the supplementary note of Burren and colleagues.^20^ We also tested whether δ differed from 0 for each feature independently and considered a trait was significant for a given feature at a FDR of less than 0.01 and suggestive at a FDR of less than 0.05. Traits were considered feature significant only if they were significant for a given feature and overall. We manually removed redundant IMD projections (i.e., multiple projections corresponding with the same or very similar disease or diagnosis) to facilitate interpretation, giving preference to datasets with larger case sizes and multi-ancestry when available.

### Distance and clustering analyses

One of our aims was to investigate the genetic relationships with other IIMs from the projections. Clustering is a useful tool to group diseases according to genetic similarity within these features. However, there are particular properties of the data that make the choice of clustering method difficult. First, there is uncertainty about each projection (captured in a standard error), and second, despite the use of principal component analysis in feature learning, the same SNPs may contribute to multiple features, meaning that there is dependence between the features. Therefore, we took two complementary approaches to cluster IIM and IMD datasets.

The Bhattacharyya distance (*D*_*B*_) measures the similarity between two distributions, considering uncertainty and the correlations across features derived from LD among the SNPs in them.^26^ Thus, we computed a covariance matrix for each projection, containing the correlation of effect sizes, and calculated *D*_*B*_ between each pair of projections. We clustered diseases using the complete linkage method in *hclust* R function and called clusters in the data using the *cutree* function with *k* = 9, as this was the largest *k* that captured an IIM cluster we observed.

However, the number of clusters cannot be reliably learned from the data in hierarchical clustering. Therefore, we also applied a Bayesian nonparametric clustering method, Dirichlet process mixtures with uncertainty (DPMUnc),^27^ that extended a standard Dirichlet process mixture model, which allows k to be estimated, to include measured uncertainty in observations. We ran DPMUnc with five parallel chains and the following parameters: kappa0 = 0.01, alpha0 = 2, beta0 = 0.1, nIts = 5,000,000, and scaleData = TRUE. After checking that all five chains converged (Figures S1 and S2), we removed the first half of each chain as burn in to avoid undue influence from initial values and summarized the remaining samples in a posterior similarity matrix (PSM). Then, we used the complete linkage method to cluster the diseases according to the PSM and used the minbinder algorithm^28^ to call clusters. Since our foci are IIMs, we used data (projection estimates and variance) from seven features for which any IIM dataset was significant at a FDR of less than 1% (PC1, 2, 3, 8, 9, 12, and 13) in both approaches.

### Colocalization and driver SNP follow-up

We investigated whether IIM associations with individual features reflected a sharing of disease causal variants between IIMs and IMDs associated with the same features.

First, we identified driver SNPs that showed evidence of association with any IIM and another IMD. The 7 IIM-associated features relate to 255 unique driver SNPs. We extracted *Z* scores for those 255 SNPs from the summary statistics of the IIM and genetically similar IMD datasets defined using clustering (see results and Table S4), computed their *p* values, and applied FDR to this set of SNPs for each disease in parallel. The FDR estimates the probability that an SNP is not associated with a given trait from its *p* value. Assuming these probabilities are independent, we can, therefore, compute the probability that a given SNP is associated with both a given IIM form and another IMD, as follows:

$$P\left(IIM\;and\;IMD\;associated|\;p_{IIM},p_{IMD}\right)=\left(1-FDR_{IIM}\right)\;\left(1-FDR_{IMD}\right)\text{,}$$

where p_IIM_ and p_IMD_ are the *p* values for the SNP with the IIM and the comparator IMD, and FDR_IIM_ and FDR_IMD_ are the estimated FDR at the same SNP for the IIM and the comparator IMD.

However, the premise behind our analysis strategy is that these probabilities are not independent, but that there is positive dependence—i.e., knowing a variant is associated with an IMD, we assume it may be more likely to also associate with IIM. Allowing for positive dependence, we see the equation above gives a lower bound, so it is a conservative estimate of the probability of joint association, since

$$P\left(IIM\;and\;IMD\;associated\;|\;p_{IIM},p_{IMD}\right)=P\left(IIM\;associated\;|\;IMD\;associated\right)\;P\left(IMD\;associated\right)\geq P\left(IIM\;associated\right)\;P\left(IMD\;associated\right)=\left(1-FDR_{IIM}\right)\;\left(1-FDR_{IMD}\right)\text{.}$$

Thus, we can calculate the probability that at least one of IIM or IMD is not associated as

$$PairwiseFDR\leq 1-\;\left(1-FDR_{IIM}\right)\left(1-FDR_{IMD}\right)\text{.}$$

Pairwise FDR summarizes evidence for association. To address the more specific hypothesis of shared causal variants, we used *coloc*.^29^ We selected all driver SNP and IMD pairs with a pairwise FDR of less than 0.05 (meaning a shared association is likely) for coloc analysis. We thinned the SNPs by distance to at most one SNP in a 1-Mb window, keeping the SNP with the minimum pairwise FDR. This resulted in 13 driver SNPs and 61 IIM-IMD pairs. Using a 2-Mb window centered at the focus SNP, we ran coloc with prior p12 = 5e−6, a conservative but robust prior according to simulations (Table S5).^30^

Coloc provides posterior probabilities (PP) across five hypotheses in each colocalization test. We are interested in hypothesis H4 (i.e., both traits have a single shared causal variant in the defined region) and considered PP H4 > 0.5 and PP H4 > 0.8 as medium and strong confidence colocalization signals, respectively. The query driver SNP might not be the shared causal SNP, so for PP H4 > 0.5 signals, we report the top candidate SNP (i.e., the SNP with the highest PP of being the shared causal variant in each region). This is analogous to fine mapping a single GWAS peak using approximate Bayes factors. Thus the top candidate SNP is the most likely causal variant, subject to specific assumptions:

(1) that the causal variant is included in the SNPs available for both traits;
(2) that there is a single causal variant in the fine mapping region; and
(3) that the assumption of colocalization holds, as these regions were selected with PP H4 > 0.8, there is a chance (1 - PP.H4) that colocalization does not hold.

Where associations within the same regions pointed at different candidate causal SNPs, we highlight the top candidate causal SNP for each region based on the PP H4 associated with it or the number of times it was assigned by coloc as the candidate SNP across associations in the same region. We queried OpenTargets Genetics to retrieve the rsIDs of the driver and candidate SNPs and the names of their nearest genes. To facilitate interpretation, when there was a neighboring gene with a better-known immunity role compared to the nearest gene, we reported it instead in tables and figures. We called novel associations those candidate SNPs that (1) have high posterior to be shared (PP H4 > 0.5) between a given IIM and an IMD, (2) are not genome-wide significant (i.e., $p\;>\;5\times 10^{-8}$) in the IIM GWAS, (3) the top hit (i.e., the one with the lowest *p* value) in the 1-Mb region defined for coloc is not genome-wide significant, and (4) was not previously reported as genome-wide significant by any IIM GWAS to our knowledge.

### Validation of approach

To estimate the chance that the above approach produces false-positive results, we replicated the process with 17 IMD traits from FinnGen Release 5 (R5)^22^ in place of the IIM traits (Table S6). The FinnGen R5 traits had case numbers from 1,290 to 22,997, a wide range of sample sizes, making them suitable for general validation of our approach. We projected each R5 trait onto the feature space, identifying significant features as for IIM and its subtypes. For each significant R5 IMD, we found the closest IMD from our IMD pool (excluding equivalent R7 IMD and IIM traits) using *D*_*B*_. As there is no one-size-fits-all threshold for *D*_*B*_, we chose the closest 17 IMD, as we did in our IIM analyses. Then, as before, we computed FDR on *p* values of driver SNPs of significant features in each R5 and their close IMD traits, and pairwise FDR for each pair. For those signals with a pairwise FDR of less than 0.05, we ran coloc as described above. For all hit variants declared by our approach (that is, a pairwise FDR of <0.05 and coloc H4 > 0.5 or 0.8), we evaluated whether the variants had smaller or larger *p* values for the same traits in FinnGen Release 10 (R10), a later release with larger sample sizes, as more patients have been added over time. Our reasoning is that small *p* values for truly null traits should, on average, have become larger, whereas those for truly associated traits should, on average, have become smaller. We explored this expectation in a simple simulation and showed that our metric—the proportion of *p* values that get smaller in the second release—varies as a function of the true proportion of false positives in a sample (Note S1 and Figure S3). However, we note that while it is related to the proportion of false positives, it is also related to the strength of association at a given SNP and does not directly quantify the proportion of false positives. We performed the same FinnGen R5 vs. R10 comparison for lead SNPs in R5 with *p* < 5 × 10^−8^ and separated by at least 1 Mb. This allowed us to compare the frequency of SNPs that validated our *p* value comparison in our approach above to those identified at the conventional genome-wide significance threshold (Table S7).

## Results

### IIM projections

We projected all 10 IIM datasets from the Miller and Rothwell studies onto the 13-dimension feature space, together with projections from a curated collection of 466 summary statistics covering a broad array of IMD (Tables S2 and S3). We tested whether the location of each GWAS trait differed from those of a null GWAS control by feature and overall, calling significance at a FDR of less than 1%. Of 476 datasets, 274 (57.6%) were significantly different from the null control overall (see Methods), including all IIM datasets (Table S1), except for the IBM subtype. The lack of significance in the projections can be explained by low statistical power due to sample size (e.g., IBM N cases = 252), or by specific IMD not sharing the factors with the IMD used to train the basis or having less clear immune involvement, resulting in a weak or null signal (Figure S4).

**Figure 1:**
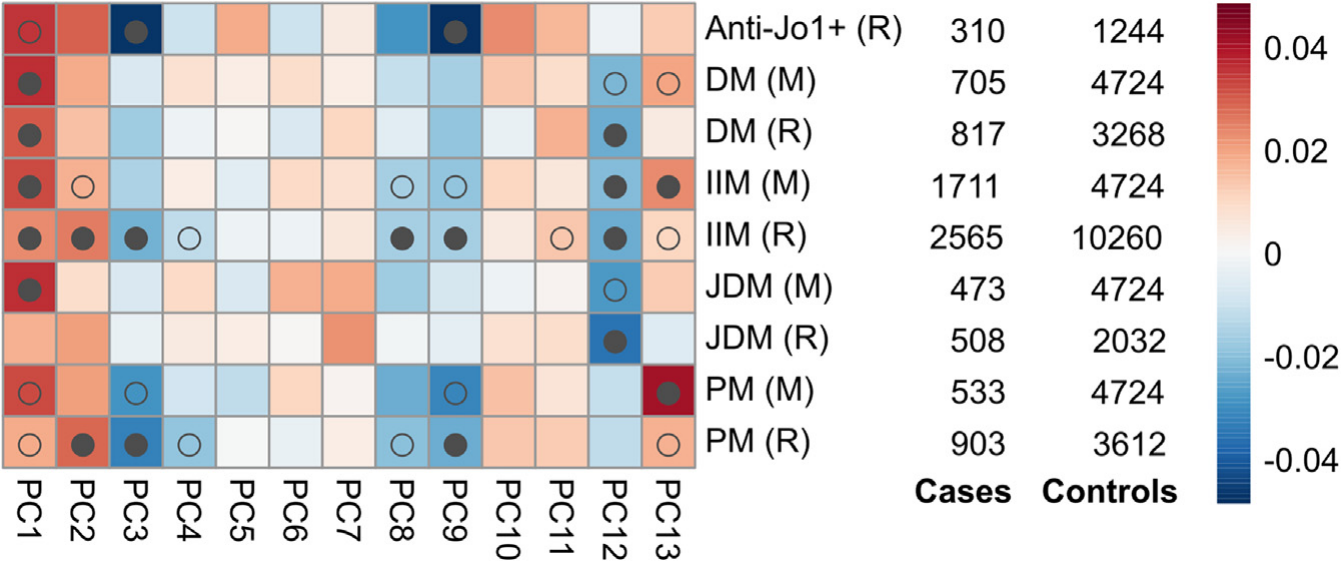
Heatmap showing overall significant (FDR <1%) IIM datasets from Miller and Rothwell studies across 13 features Colors represent projection values; similar colors mean the projections are closer in a given feature. Full dots represent the dataset is significant for the feature at a FDR of less than1%, and hollow dots represent significance at a FDR of less than 5%. For 7 of 13 features, at least 1 myositis dataset was significant at a FDR of less than 1%. IIM (M)/(R), meta-analyses; M, Miller; R, Rothwell.

At the feature level, projections of at least one IIM dataset showed significant associations to 7 of 13 features at a FDR of less than 1%: PC1-3, PC8-9, and PC12-13 (Figure 1). Projections of IIM are significant for more features at a FDR of less than 1% than subtype projections, and likewise for Rothwell datasets compared to Miller datasets, as expected given the larger sample sizes in the former.

Some IIM-associated features have established interpretations, allowing us to characterize IIM biological aspects. For example, PC1 distinguishes autoimmune (positive, red) from autoinflammatory (blue, negative), and all IIM datasets are associated with the former, reflecting its autoimmune nature. IIMs are also associated with higher expression of the interferon-driven chemokines CXCL9 (MIG) and CXCL10 (IP-10), captured by negative PC3, and with increased eosinophil counts, associated with positive PC13. The other four features associated with IIM (PC2, 8, 9, and 12) are not yet biologically characterized (Figures S5–S11). However, we can appreciate some patterns. For example, PC2 seems to be SLE dominated, with PM and other connective tissue diseases on the same side. PC9 features rheumatoid arthritis (RA) and multiple sclerosis on opposite extremes, with Anti-Jo1^+^, PM, and other IMD like PR3^+^ ANCA-associated vasculitis (AAV) and hyperthyroidism (HyperThy) on the same side as RA.

### IIMs in the context of other IMDs

**Figure 2:**
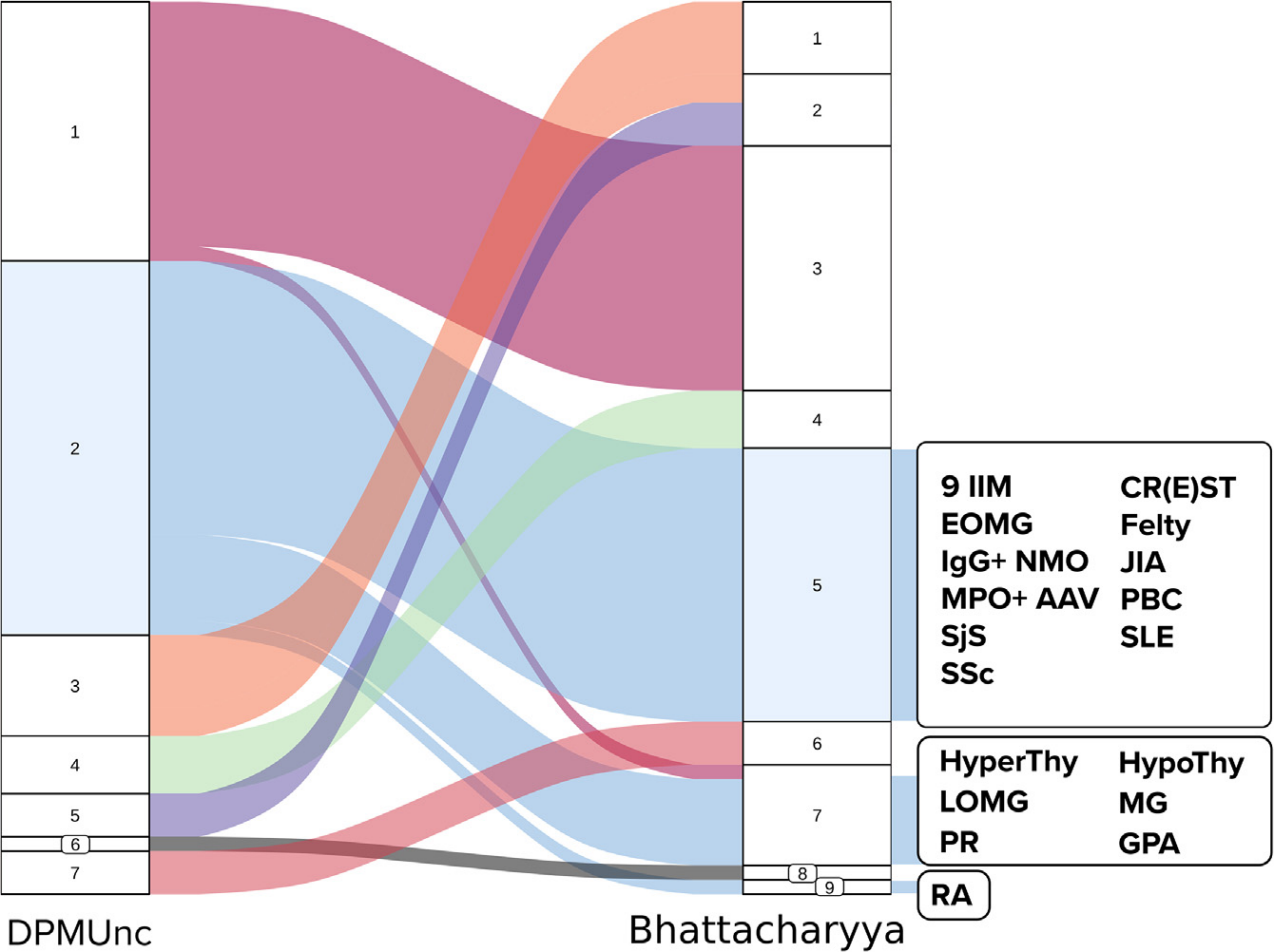
Relationships among DPMUnc and *D*_*B*_ clusterings DPMunc called seven clusters, while we called nine using *D*_*B*_. IIM datasets are allocated to cluster 2 in DPMUnc and cluster 5 in *D*_*B*_, along with other IMDs (highlighted in light blue). Other IMDs in DPMUnc cluster 2 are located in *D*_*B*_ clusters 7 and 9. CR(E)ST, CR(E)ST syndrome; Felty, Felty syndrome; IgG^+^ NMO, IgG^+^ neuromyelitis optica; JIA, juvenile idiopathic arthritis; LOMG, late-onset myasthenia gravis; PR, palindromic dermatitis.

To explore the relationships between IIMs and other IMDs in our collection, we filtered the 274 overall significant IMD datasets to remove redundant traits, resulting in 62 IMD projections (9 IIM + 53 IMD) (Table S2). Clustering using the *D*_*B*_ placed IIM and all its subtypes in the same cluster, together with 10 other IMDs: CR(E)ST syndrome (MIM: 181750), early-onset myasthenia gravis (EOMG [MIM: 254200]), Felty syndrome (MIM: 134750), IgG^+^ neuromyelitis optica, juvenile idiopathic arthritis (MIM: 618795), MPO^+^ AAV (MIM: 608710), primary biliary cholangitis (PBC), SjS, SLE, and SSc (Figures 2 and S12).

DPMUnc assigned all diseases in the Bhattacharyya IIM cluster to the same cluster but also included seven other IMD. This larger IIM cluster additionally included hypothyroidism (HypoThy [MIM: 140300]) and HyperThy (MIM: 275000), late-onset and pooled MG (EOMG and MG), palindromic rheumatism (PR), RA (MIM: 180300), and granulomatosis with polyangiitis (also known as Wegener’s granulomatosis [GPA] [MIM: 608710]) (Figure S13). Given that the clustering using the *D*_*B*_ required us to pick the number of clusters, while the DPMUnc method can choose them automatically, and the similarity between the solutions, we decided to consider any IMD co-clustering by either method as genetically related to IIM.

### Identifying SNPs connecting IIM with other IMD

We investigated whether we could leverage the genetic similarity of IIMs to these other IMDs with often larger datasets to identify novel IIM genetic associations.

**Figure 3:**
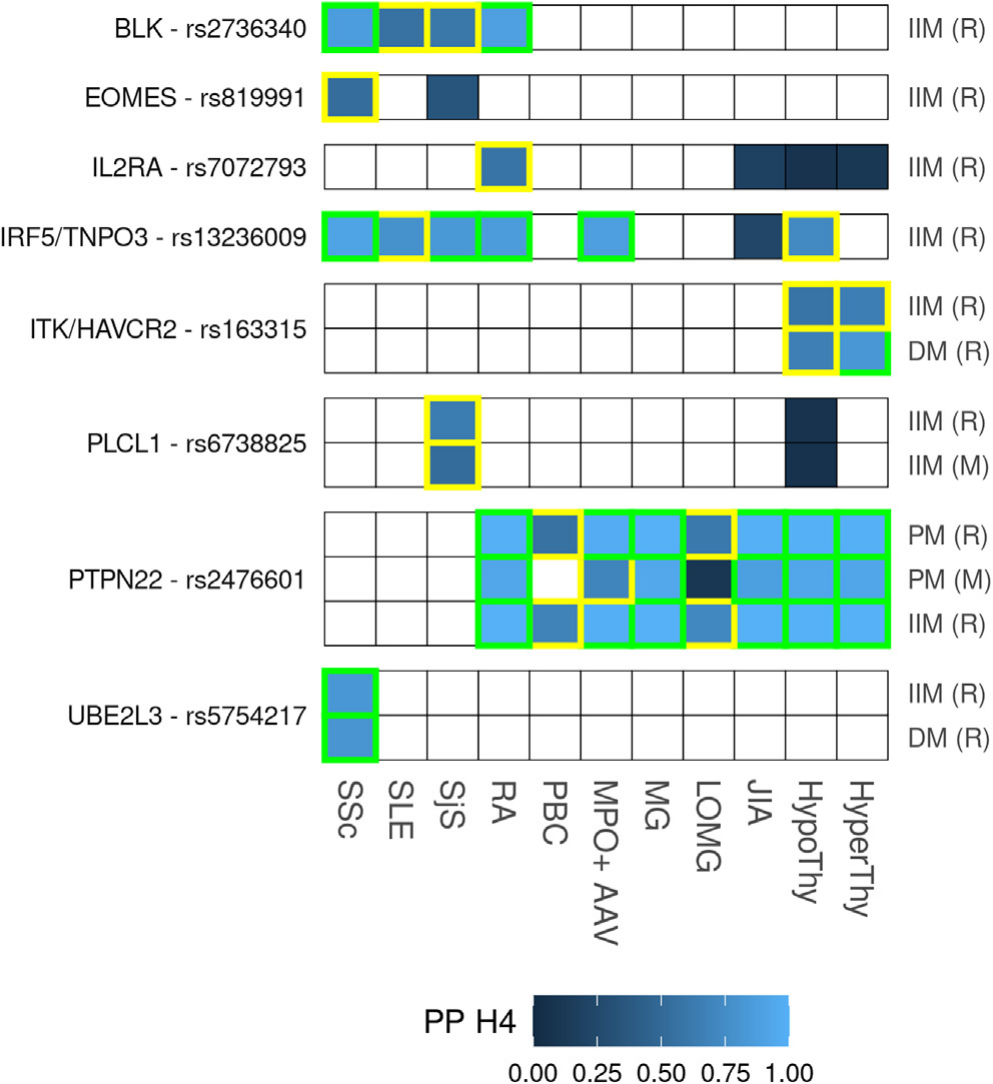
PPs for shared causal variants (H4) between IIM and selected IMDs at eight top candidate SNPs A colored square indicates the colocalization analysis was performed, and the shade of color represents the PP H4 for the test. Medium confidence associations (0.5 < PP ≤ 0.8) are marked in yellow rectangles and high confidence (PP > 0.8) associations in green rectangles. Top candidate SNPs are labeled with their nearest gene or with a nearby strong IMD candidate gene when we find one. JIA, juvenile idiopathic arthritis; LOMG, late-onset myasthenia gravis. (M) and (R) represent that the dataset comes from the Miller or Rothwell study, respectively. IIM labels represent meta-analysis datasets.

We selected all 17 IMDs identified as genetically similar to IIM at the clustering step to explore specific associations with each of the nine Miller and Rothwell IIM datasets at the SNP level (See Table S4). The genetic features are linear functions of summary effects at a small set of SNPs, referred to as driver SNPs. A screening approach based on pairwise FDR identified 13 driver SNPs as likely to be associated with both a IIM trait and another IMD (61 IIM-IMD pairs). We used coloc^29^ to formally investigate whether these associations correspond with shared putative causal variants or distinct but neighboring causal variants. Regions around 8 of 13 SNPs showed evidence of shared causal variant association (PP H4 > 0.5) between at least 1 IIM and a selected IMD dataset as well as a pairwise FDR of less than 0.05, with 5 of the 8 driver SNPs having strong evidence (PP H4 > 0.8). Finally, seven signals were novel (i.e., no reported genome-wide significant association in the region in any IIM dataset in our study or other publications) (Figure 3 and Tables 1 and S5).

**Table 1:** Colocalization results from 8 SNPs with support for a shared causal variant (PP H4 > 0.5) and pairwise FDR of <0.05 for at least one IIM/IMD pair

Driver SNP	Top candidate SNP	Open targets candidate gene	Top SNP *p*-value^a^	IIM	IMD	Pairwise FDR	H4
rs13277113	rs2736340^b^	*BLK*	1.92E−04	IIM (R)	RA	0.0046	**0.8830**
					SLE	0.0045	0.5561
					SSc	0.0044	**0.8667**
					SjS	0.0045	0.5988
rs669607	rs819991^b^	*EOMES*	1.06E−03	IIM (R)	SSc	0.0343	0.5254
rs7072793	rs7072793^b^	*IL2RA*	1.19E−03	IIM (R)	RA	0.0226	0.5754
rs10488631	rs13236009^b^	*IRF5/TNPO3*	6.41E−05	IIM (R)	HypoThy	0.0039	0.7340
					MPO+ AAV	0.0044	**0.8800**
					RA	0.0039	**0.8581**
					SLE	0.0039	0.7847
					SSc	0.0039	**0.9105**
					SjS	0.0039	**0.8356**
rs394378	rs163315^b^	*ITK/HAVCR2*	3.78E−05	DM (R)	HyperThy	0.0361	**0.8199**
				DM (R)	HypoThy	0.0359	0.6617
			6.22E−04	IIM (R)	HyperThy	0.0145	0.6554
					HypoThy	0.0143	0.5699
rs10196612	rs6738825^b^	*PLCL1*	1.16E−04	IIM (M)	SjS	0.0327	0.5245
			3.10E−05	IIM (R)	SjS	0.0046	0.6385
rs2476601	rs2476601	*PTPN22*	1.63E−07	IIM (R)	HyperThy	0.0000	**0.9979**
					HypoThy	0.0000	**0.9975**
					JIA	0.0000	**0.9975**
					LOMG	0.0186	0.7147
					MG	0.0000	**0.9985**
					MPO+ AAV	0.0007	**0.9849**
					PBC	0.0460	0.6795
					RA	0.0000	**0.9975**
			2.59E−05	PM (M)	HyperThy	0.0129	**0.9272**
					HypoThy	0.0129	**0.9313**
					JIA	0.0129	**0.8806**
					MG	0.0129	**0.9417**
					MPO+ AAV	0.0136	0.6967
					RA	0.0129	**0.9311**
			1.25E−06	PM (R)	HyperThy	0.0001	**0.9957**
					HypoThy	0.0001	**0.9952**
					JIA	0.0001	**0.9942**
					LOMG	0.0187	0.6055
					MG	0.0001	**0.9967**
					MPO+ AAV	0.0008	**0.9734**
					PBC	0.0461	0.5613
					RA	0.0001	**0.9952**
rs5754217	rs5754217^b^	*UBE2L3*	3.00E−04	DM (R)	SSc	0.0372	**0.8055**
			7.44E−05	IIM (R)	SSc	0.0053	**0.8255**

Results with PP H4 > 0.8 are highlighted in bold.

(M) and (R) represent the Miller and Rothwell studies, respectively. IIM (R)/(M) labels represent meta-analyses

a For each test, coloc reports the SNP with the highest PP to be the causal in the region. Since these signals are likely to be caused by one rather than multiple SNPs, here we report the top candidate SNP *p*-value (in the IIM dataset), selected for being associated with the highest PP H4 or for being identified as the candidate SNP in most tests.

b Novel signals.

Most colocalization signals come from the four connective tissue diseases mentioned above that co-occur most commonly with IIM: RA, SLE, SjS, and SSc. The driver SNP with the most colocalizations between IIM and IMD is rs2476601, a missense variant of *PTPN22* and one of the best-known non-MHC risk variants associated with autoimmune disease. Our results show high confidence of colocalization at rs2476601 between IIM (meta-analyses)/PM and multiple autoimmune diseases, but no colocalization signals in anti-Jo1^+^, DM, or JDM (Figure 3), which suggests the signal we observe in the IIM for this SNP may come predominantly from PM patients, although a similar magnitude though less significant signal is also seen for anti-Jo1^+^ myositis (Figure S14). The lack of colocalization signal for anti-Jo1^+^ may reflect lower power due to sample size rather than lack of association. *PTPN22* encodes LYP, a negative regulator of T cell receptor signaling. Several other colocalizations also implicate T cells in IIM.

rs163315 indexed medium- and high-confidence colocalization of DM and IIM with HyperThy and HypoThy. This variant lies in an intron of *ITK*, another tyrosine-protein kinase highly expressed in T cells,^31^ which promotes pro-inflammatory Th17 differentiation.^32^ However, rs163315 is also an eQTL for *HAVCR2*, which encodes TIM3, an inhibitory receptor for which loss of function mutations lead to a severe autoinflammatory and autoimmune phenotype.^33^

rs7072793 indexed medium confidence (PP H4 = 0.57, pairwise FDR = 0.02) colocalization between IIM and RA in the *IL2RA* region. IMD association with *IL2RA* is known to be complex, with multiple causal variants in the gene.^34^ Thus, rs7072793 may be tagging multiple IIM signals in the region, but the strength of the signal in IIM alone precludes fine mapping. IMD-associated variants in this region have been shown to decrease interleukin-2 signaling and decrease regulatory T cell (Treg) function.^35^

rs819991 indexes a medium confidence (PP H4 = 0.53, pairwise FDR = 0.03) colocalization between IIM and SSc. This intergenic variant is an eQTL of *EOMES*, which encodes eomesodermin, a transcription factor with a role in CD8^+^ Treg homeostasis.^36^

rs5754217 indexes strong colocalizations (PP H4 = 0.80–0.82) between DM/IIM and SSc. This candidate SNP is an intron variant and an eQTL of *UBE2L3* (ubiquitin-conjugating enzyme E2 L3), which participates in the ubiquitination of many target proteins and regulates pathways like the NF-κB.^37^ *UBE2L3* has been previously reported to be associated with increased risk for SLE,^38^^,^^39^^,^^40^ Crohn disease (MIM: 266600),^41^ and HypoThy (where rs5754217 was identified as one of the risk variants),^42^ among other IMDs.

Other colocalization hits are specifically B cell related. rs2736340 indexed strong colocalization signals of IIM with RA and SSc and weaker for SLE and SjS. rs2736340 has been previously identified as a risk variant for multiple IMDs,^43^ including DM in an early GWAS,^7^ but was not genome-wide significant in the two IIM studies considered here. Immune disease risk variants have been linked to a lower expression of the nearest gene *BLK* and lower thresholds for B cell receptor signaling.^44^

rs13236009 showed strong colocalization signals for IIM with MPO^+^ AAV, RA, SjS, and SSc and weaker signals for HypoThy and SLE. This variant is an intron variant of *TNPO3*, of which it is also an eQTL and is a novel candidate SNP for IIM. *TNPO3* encodes a nuclear import receptor and is physically proximate to *IRF5*, a regulatory factor that regulates the expression of interferon and is critical to many inflammatory pathways. Many variants in the *IRF5-TNPO3* locus have been associated with SLE, SjS, SSc, PBC, and other IMD.^45^^,^^46^^,^^47^^,^^48^

rs6738825 indexes a medium confidence (PP H4 = 0.52–0.63) colocalization between IIM and SjS. This is an intron variant and an eQTL of *PLCL1* (phospholipase C-like 1). This protein has been previously identified as a risk locus for RA, as it regulates inflammation of fibroblast-like synoviocytes in RA patients.^49^ In addition, rs6738825 has been identified as one of the modulators of allergic rhinitis susceptibility (in a Chinese population).^50^

### Validation of approach

**Figure 4:**
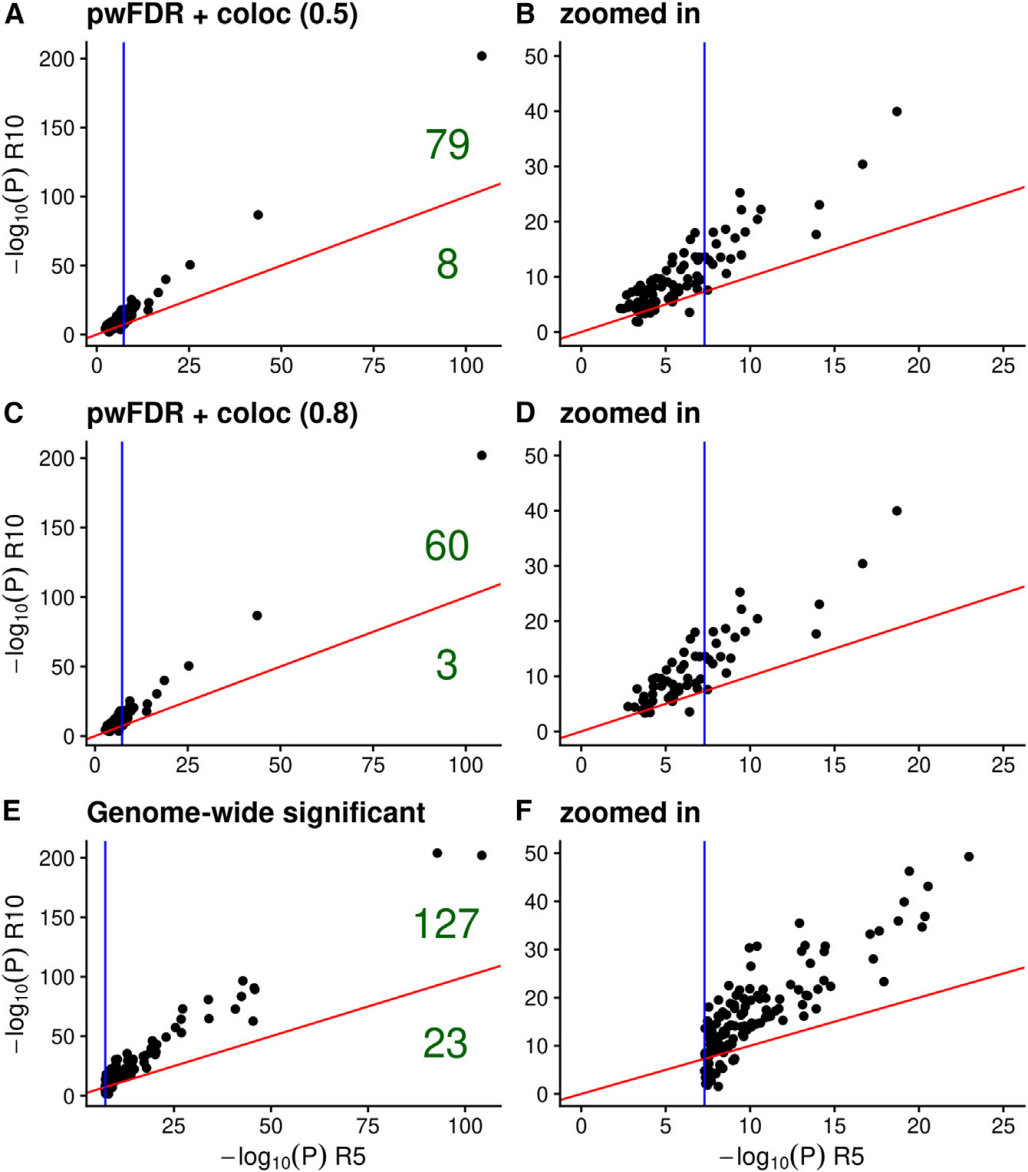
Comparison of *p*-values of SNPs from FinnGen R5 and FinnGen R10 used in our validation process (A and C) SNPs identified using our pairwise FDR and colocalization approach (pwFDR + coloc) at two PP H4 levels (H4 > 0.5 and H4 > 0.8). (E) SNPs identified using a conventional genome-wide significant threshold (P < 5e−8). (B, D, and F) Zoomed-in view of the data showed in (A, C, and E). The diagonal red line represents the validation threshold (−log10(P) R5 = −log10(P) R10). The vertical blue line represents the genome-wide significant threshold. Numbers in green represent the number of SNPs that validate (above red line) and those that do not (below red line).

When we performed the same set of analyses on 17 IMD traits from FinnGen revision 5 (Table S6), we identified 63 (87) trait-variant associations with a pairwise FDR of less than 0.05 and coloc PP H4 > 0.8 (0.5). We calculated that for 4.76% (9.2%) of these, *p* values became larger between FinnGen R5 and R10 (Table S7). While we expect *p* values of truly associated SNPs to get smaller in larger samples (and vice versa for truly null SNPs), this is not guaranteed, particularly for diseases with relatively small case counts. Thus we do not expect this ratio of larger/smaller *p* values to be 0, even if all effects detected are true. For these same R5 traits, we found 150 genome-wide significant SNPs at least 1 Mb apart, of which 15.33% *p* values became larger in R10. This suggests that our approach produces results with a similar FDR to using a genome-wide significance threshold, but it is helpful at finding SNPs below the genome-wide significant threshold, which makes it useful to find novel causal variants in smaller GWASs (Figure 4).

## Discussion

IIMs are rare and heterogeneous disorders that are often challenging to classify and assign patients to upon clinical diagnosis. Here, we used data from two GWAS that provided data on five IIM subtypes in combination with a collection of selected IMD datasets, allowing us to both distill the genetic basis of the subtypes and compare the signals found in the data across both studies. While the signals found in IIM subtypes generally agree in direction, we observed differences in the intensity of the signals. For example, we find the strongest autoimmune signal in DM and JDM (PC1), whereas anti-Jo1^+^ has its strongest signal with the *CXCL9/CXCL10* feature (PC3) and uncharacterized PC9, and PM is most strongly associated with eosinophil levels (PC13) (Figure 1). However, although there is suggestive evidence that the *PTPN22* signal is specific to PM^11^ and anti-Jo1^+^, no two IIM subtypes differed significantly in their location on any of these features. This may reflect a lack of power, but also emphasizes a degree of commonality of genetic risk between subtypes (Figure S15).

Regarding specific feature-IIM associations, previous reports have shown *CXCL9* and *CXCL10* to be upregulated in IIM, with elevated levels of both cytokines in muscle and serum, in concordance with the signals observed in PC3.^51^^,^^52^ Elevated levels of both chemokines have also been associated with anti-Jo1^+^ antibodies in patients with interstitial lung disease, a common complication of IIM.^53^ Elevated CXCL10s have also been identified among the biomarkers of disease activity in JDM,^54^^,^^55^^,^^56^^,^^57^ but we observe only weak and non-significant signals for JDM or DM on PC3. While elevated eosinophil levels are a hallmark of atopic IMD, like asthma,^58^ data on the role of eosinophil abundance in IIM are scarce. We found a strong eosinophilic signal associated with PM at PC13. Although very rare subtypes of IIM are characterized by elevated eosinophils (e.g., eosinophilic myositis),^59^ they are likely too rare to be driving this signal, suggesting a potential role for eosinophils in IIM, and particularly in PM.

By clustering the projections of IIM together with other IMD in our collection using two complementary methods, we identified 17 IMD with closer genetic profiles to IMD among a pool of 53 selected IMD. Six IMD (CR(E)ST, EOMG, Felty, GPA, IgG^+^ NMO, and PR) clustered with IIM by at least one method, but we could find no pairwise FDR/colocalization signals, likely due to lack of power, as these disease datasets tended to have fewer cases than for the others.

Some co-clustering IMDs reflect known comorbidities (also called overlap myositis),^60^^,^^61^ such as the connective tissue disorders RA, SLE, SjS, and SSc, revealing a partially shared genetic architecture among these IMDs. Others have a less clear relationship to IIM, such as subtypes of AAV, another rare systemic disease with heterogeneous clinical manifestations that affect small vessels.^62^ Co-occurrence between IIM and AAV is considered exceedingly rare, with only a few cases reported of patients presenting both conditions.^63^^,^^64^

MG is another disease affecting muscles and both its subtypes (early and late onset) clustered as close to IIM. Although its co-occurrence with IIM has been described only rarely in case series,^65^ both have a strong auto-antibody profile, and a review of MG cases in Swedish registry data found co-occurrence of MG with DM or PM was significantly higher than expected by chance, with an odds ratio of 21.^66^ Increased co-occurrence was attributed to the common genetic risk factor *HLA-B8-DR3*, but as the MHC region was excluded here, our results suggest a broader genetic relationship between MG and IIMs.

We also identified the two main forms of thyroid disease (HyperThy/Graves’ disease and HypoThy/Hashimoto’s disease). Muscle is a major target of the thyroid hormone,^67^ and patients with thyroid dysfunction commonly present musculoskeletal complaints and conditions, like thyrotoxic and HypoThy myopathy, frequently after treatment onset.^68^^,^^69^^,^^70^ Cases of HyperThy/HypoThy in IIM patients have also been reported, with one study identifying up to 5.5% of IIM patients developing some form of thyroid disease,^71^ in line with prevalence in the general population. Our findings suggest the genetic relationship between these IMD might be closer than previously appreciated.

Our work has some limitations. First, in both the feature engineering and colocalization processes, we assumed a single causal variant per disease and genomic region, which is unrealistic and may prevent us from finding additional causal variants, although it allowed us to analyze multiple GWAS summary statistics without accurate LD estimations. Second, we excluded the MHC region in this study, which is key to immune response and autoimmunity, and the strongest hits in almost all autoimmune GWASs are found in this region, including IIMs. By excluding this important region, we are restricting our view of shared IMD genetics. However, the MHC has a long and complex LD structure, which makes analyzing MHC signals challenging with current methods, and the strength and diversity of GWAS signals mean it might dominate the features. Its exclusion means that results here relate to genetic variants outside the MHC, which may complement results from other MHC-focused studies, such as seen with the relationship between IIM and MG. Third, four of the seven relevant genetic features for IIM lack a clear interpretation, which prevents us from further understanding some genetic aspects of IIM. Fourth, while our method has proven to capture genetic signals even in low sample size datasets, the rarity of IIM means all studies have relatively modest sample sizes, as well as the reliance on genomic imputation quality (in the Rothwell study), may limit our power to detect additional genetic signals, especially in the smallest IIM subtypes, like IBM. Finally, our approach increases discovery through learning from larger studies of related diseases. This means additional risk variants can only come from those with shared effects with other IMD and cannot reveal IIM-specific risk variants. The initial studies used to define the features may thus limit discovery. Our studies were chosen for large sample sizes and to cover a breadth of IMD. If, for example, we wanted to distinguish effects specific to JDM from DM, we may have to prioritize including a mixture of child- and adult-onset IMDs. From our initial studies, only type 1 diabetes^72^ was predominantly childhood onset, with cases coming from the UK GRID study which reports disease onset before 16 years of age as an inclusion criterion.

This work extends the genetic feature engineering work proposed by Burren et al.^20^ by using a combination of pairwise FDR and coloc to prioritize new associations, and represents a paradigm for enhanced discovery in less common diseases.

Validation analysis in FinnGen suggests that our approach has a FDR comparable with applying a genome-wide significance threshold to diseases with modest case counts. By exploiting the patterns of shared genetic architecture across common and rare IMD, we found seven novel IIM variants not found by earlier studies, an increase of more than 140% on published genome-wide significant variants outside the MHC. While our focus was on IIMs in this study, the approach is directly applicable to other IMD using the same published features we developed. It is also expandable beyond IMD, as features can be trained in any group of diseases and biological traits, such as metabolic or psychiatric traits. We proposed that leveraging information from related traits can be a powerful tool to enhance genetic discovery in rare diseases.

## Data and code availability

The datasets and code generated during this study are available its dedicated GitHub repository (see Web Resources). All publicly available GWAS summary statistics from which data for this study was derived are referenced in Tables S2 and S6.

## Web resources

GitHub repository for this project: https://github.com/GRealesM/myositis-IMD.

OpenTargets: https://genetics.opentargets.org/

GWAS_tools summary statistics processing pipeline: https://github.com/GRealesM/GWAS_tools.

Cupcake R package: https://github.com/ollyburren/cupcake.

UCSC Genome browser: http://genome.ucsc.edu/

FinnGen: https://www.finngen.fi/

Pan-UKBB: https://pan.ukbb.broadinstitute.org.

Biobank Japan: https://biobankjp.org/en/

Online Mendelian Inheritance in Man: https://www.omim.org/

## Supplemental information

Document S1. Figures S1–S15, and Note S1

Data S1. Table S1–S7

Document S2. Article plus supplemental information
