# Supplementary material for: Discovery of new myositis genetic associations through leveraging other immune-mediated diseases
Source: HGG Adv. 2024 Jul 22;5(4):100336. doi: 10.1016/j.xhgg.2024.100336 (PMC11350499; doi:10.1016/j.xhgg.2024.100336)
Supplement: Document S1. Figures S1–S15, and Note S1 [file mmc1.pdf]

HGGA, Volume *5*

Supplemental information

Discovery of new myositis genetic associations through leveraging other immune-mediated diseases

Guillermo Reales, Christopher I. Amos, Olivier Benveniste, Hector Chinoy, Jan De Bleecker, Boel De Paepe, Andrea Doria, Peter K. Gregersen, Janine A. Lamb, Vidya Limaye, Ingrid E. Lundberg, Pedro M. Machado, Britta Maurer, Frederick W. Miller, Øyvind Molberg, Lauren M. Pachman, Leonid Padyukov, Timothy R. Radstake, Ann M. Reed, Lisa G. Rider, Simon Rothwell, Albert Selva-O'Callaghan, Jiri Vencovský, Lucy R. Wedderburn, Myositis Genetics Consortium, and Chris Wallace

Supplemental Information

Table of Contents

Supplementary Figures.. 1
Supplementary Notes...14

Supplementary Figures

Figure S1 | Median trace plot of the DPMUnc run using 5 chains. All chains converged.

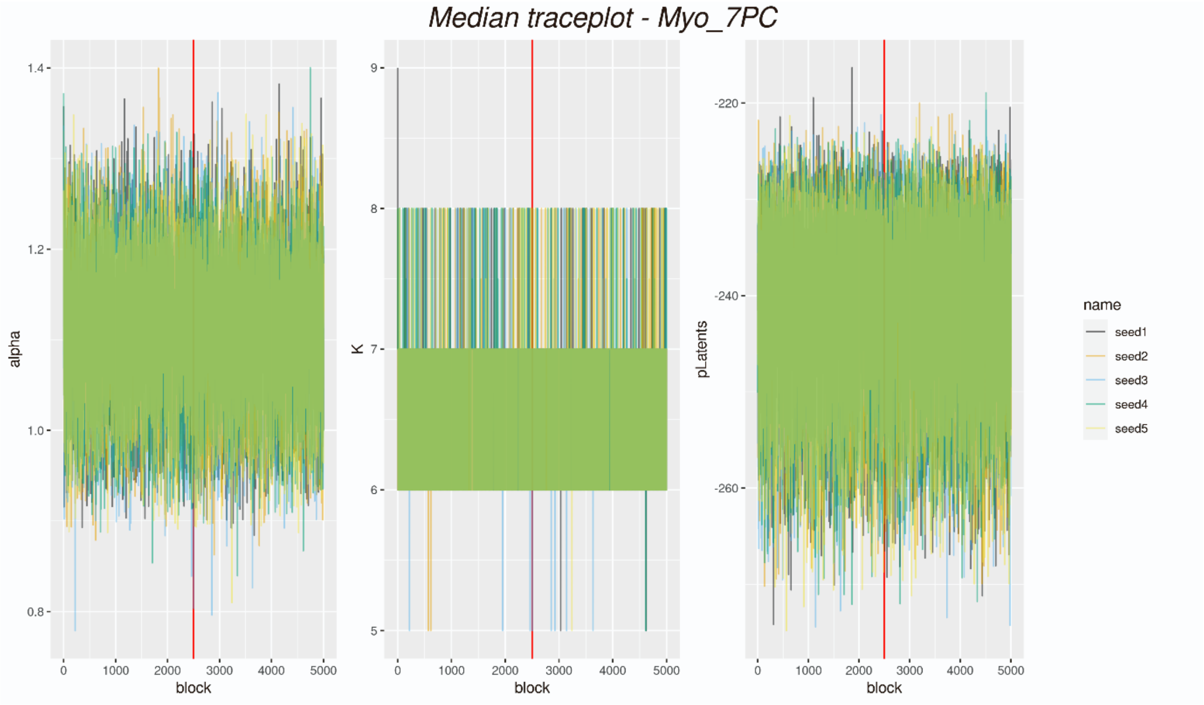

Figure S2 | Quantile trace plot of the DPMUnc run using 5 chains. All chains converged.

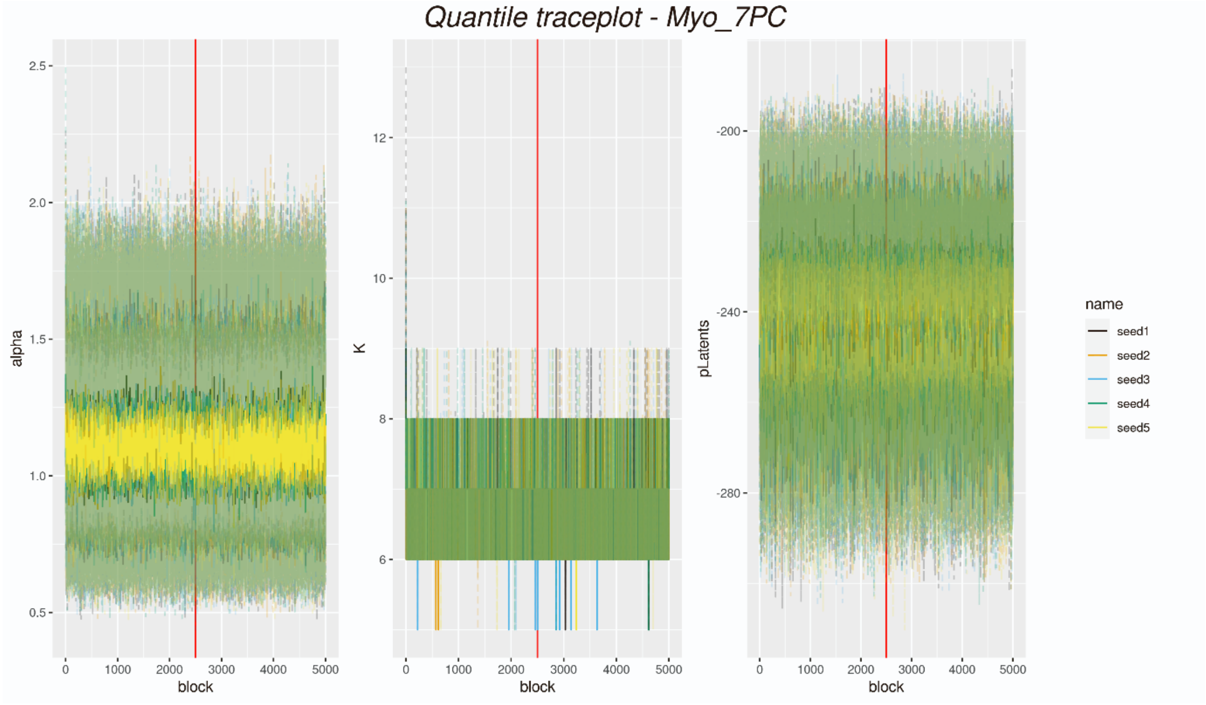

Figure S3 | An exploration of false positive rates using a simulation of a genetic study with second round data. A. P values of simulated SNPs in Round 1 (p1) and Round 1 + 2 (p2) in high power, low power, and null scenarios. B. Same as A, but including significant SNPs (p1 < 0.05) in Round 1 only. C. Proportion of p2 < p1 across mixture scenarios with different proportions of high- and low-power, and false positive rates.

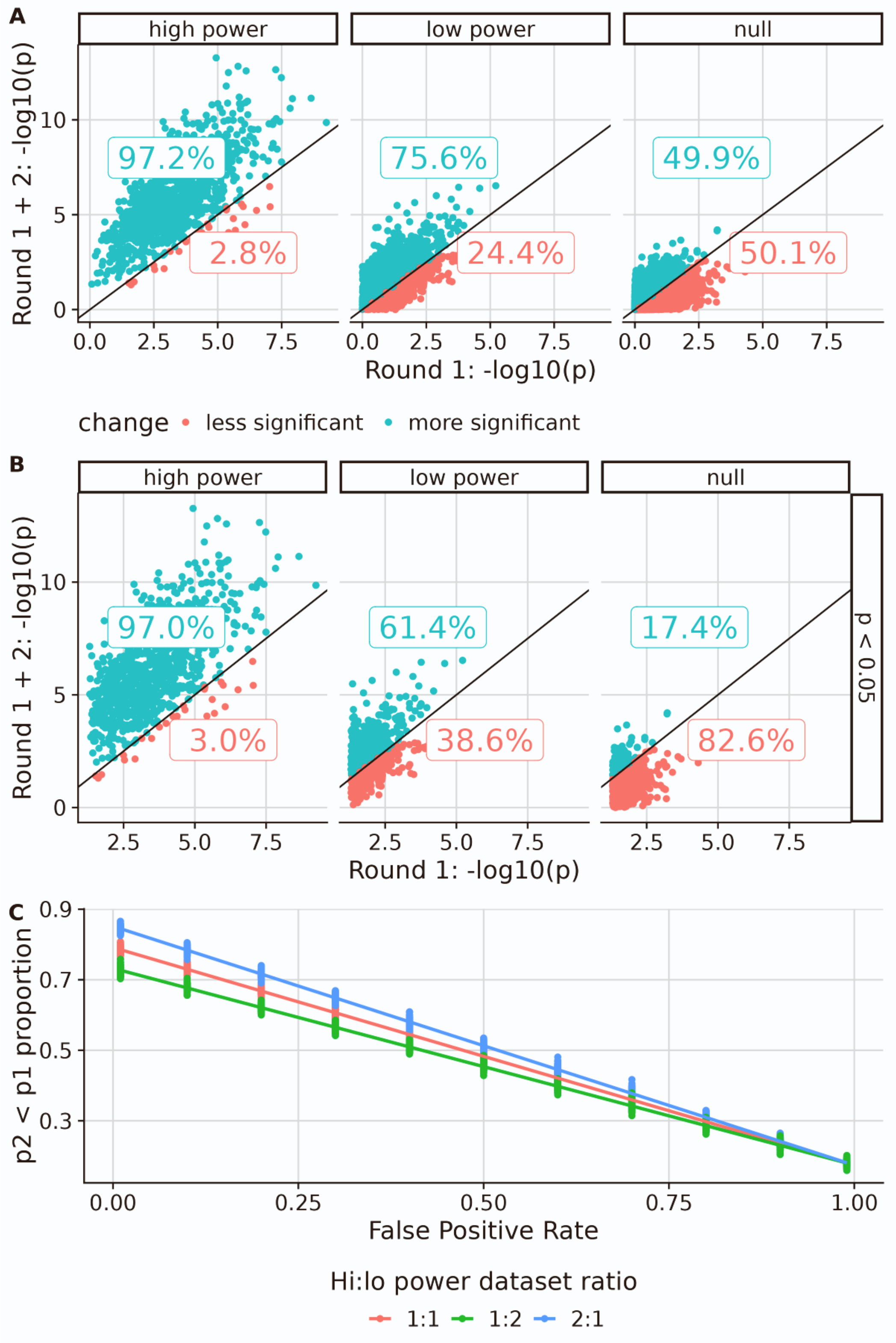

Figure S4 | Scatterplot of -log10(FDR overall) of 476 projections and their respective case numbers (N1) with IIM datasets highlighted in dark red and FDR 1% threshold represented as a dashed line. A. All projections. B. Zoomed-in version of the plot.

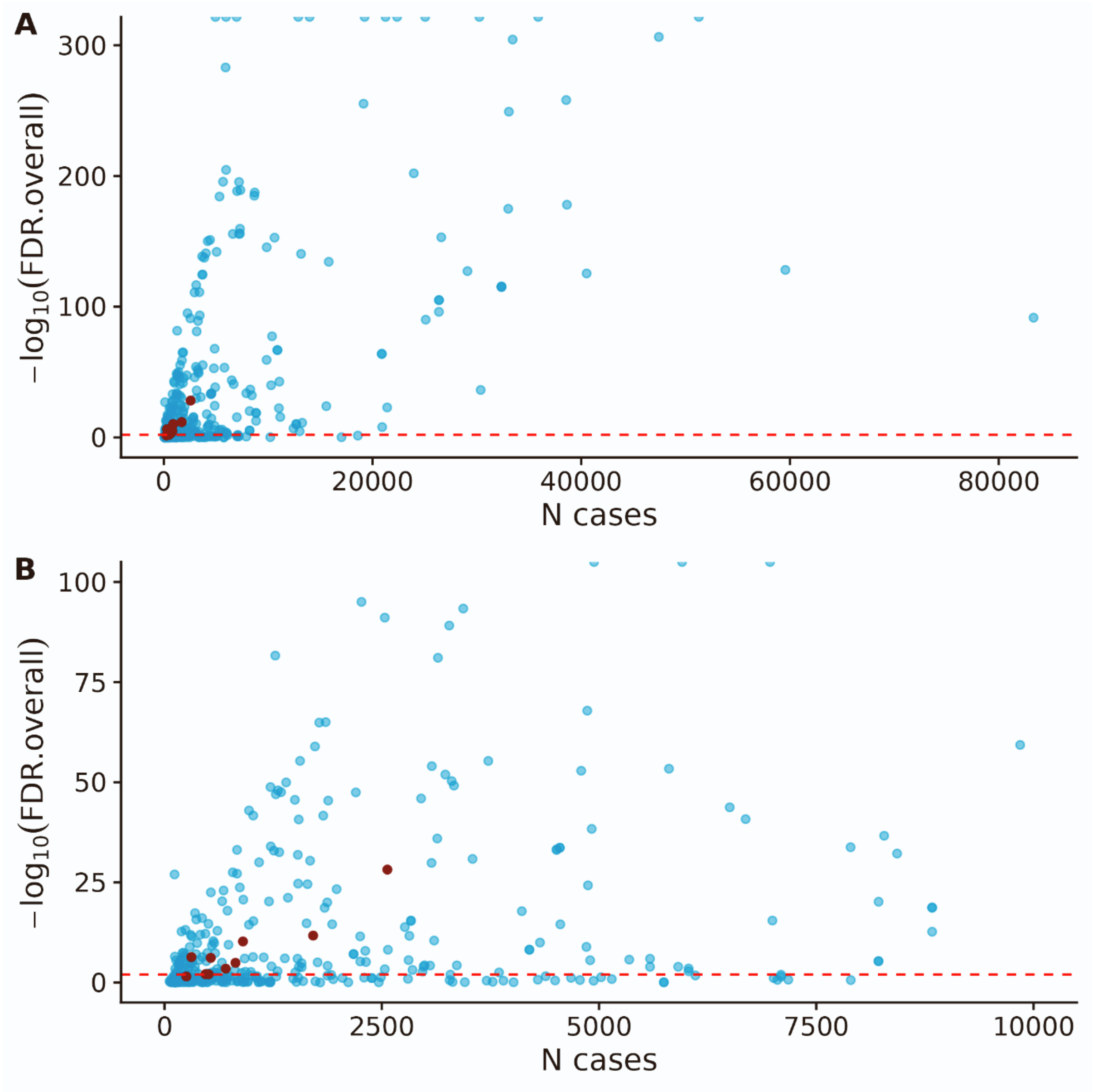

Figure S5 | Delta scores of selected feature-significant IMD at FDR 1% on PC1. Significant myositis datasets are shown in red and IMD in blue.

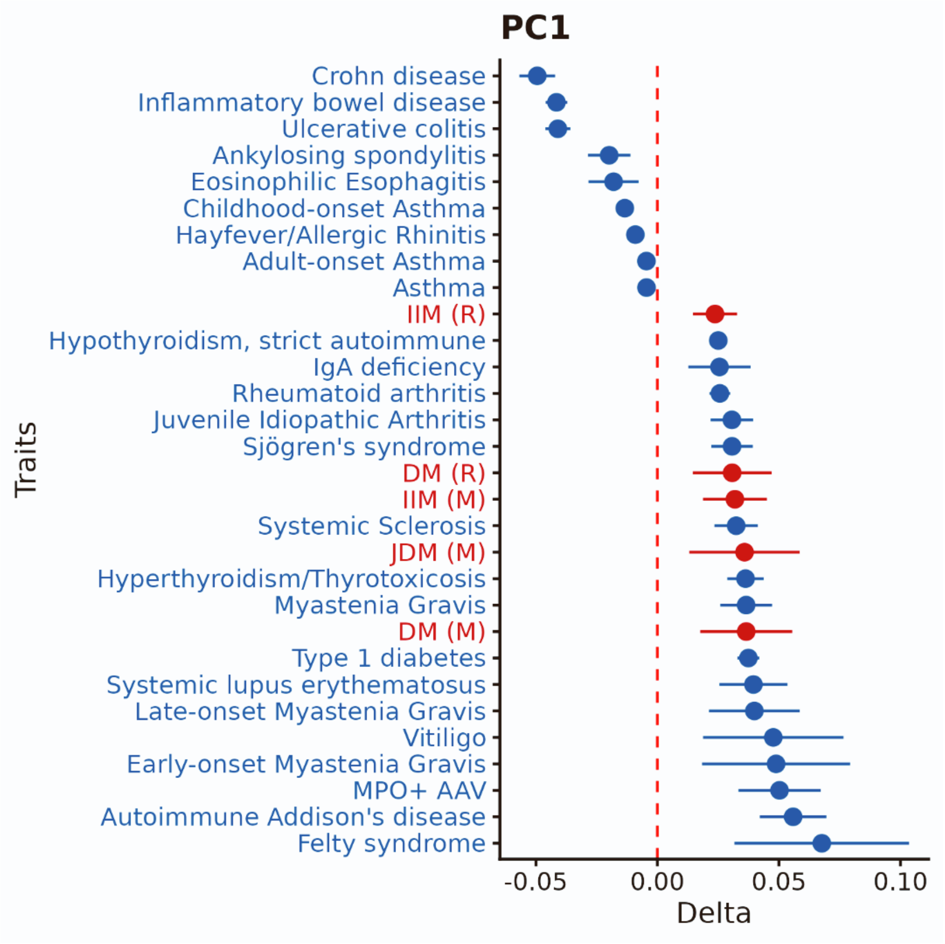

Figure S6 | Delta scores of selected feature-significant IMD at FDR 1% on PC2. Significant myositis datasets are shown in red and IMD in blue.

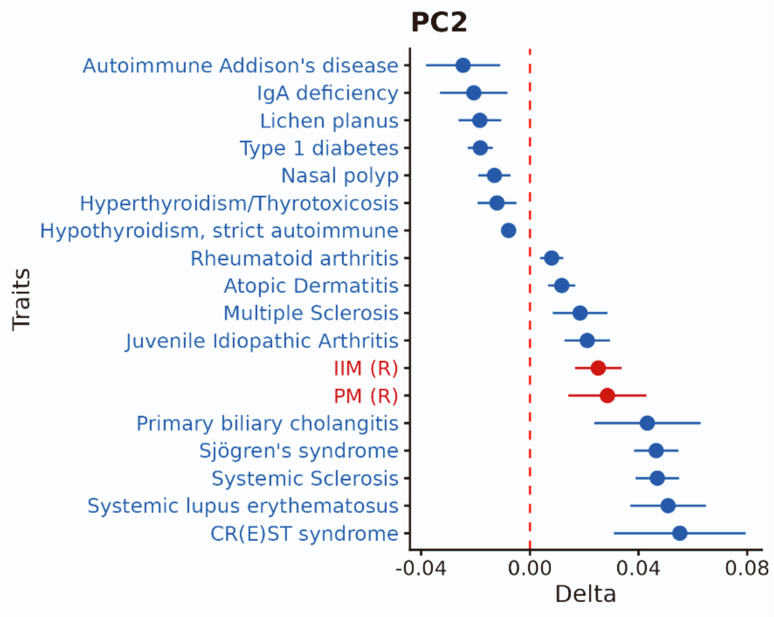

Figure S7 | Delta scores of selected feature-significant IMD at FDR 1% on PC3. Significant myositis datasets are shown in red and IMD in blue.

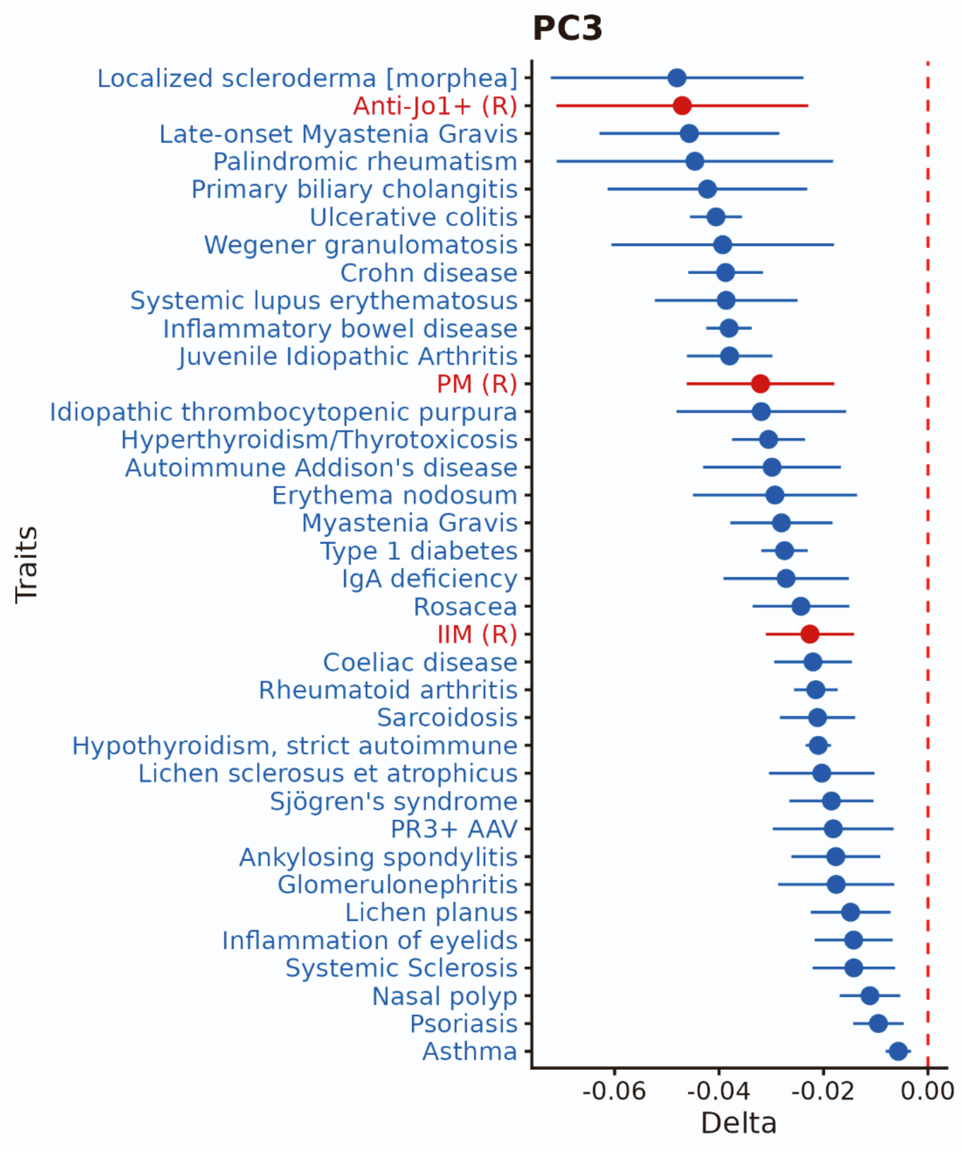

Figure S8 | Delta scores of selected feature-significant IMD at FDR 1% on PC8. Significant myositis datasets are shown in red and IMD in blue.

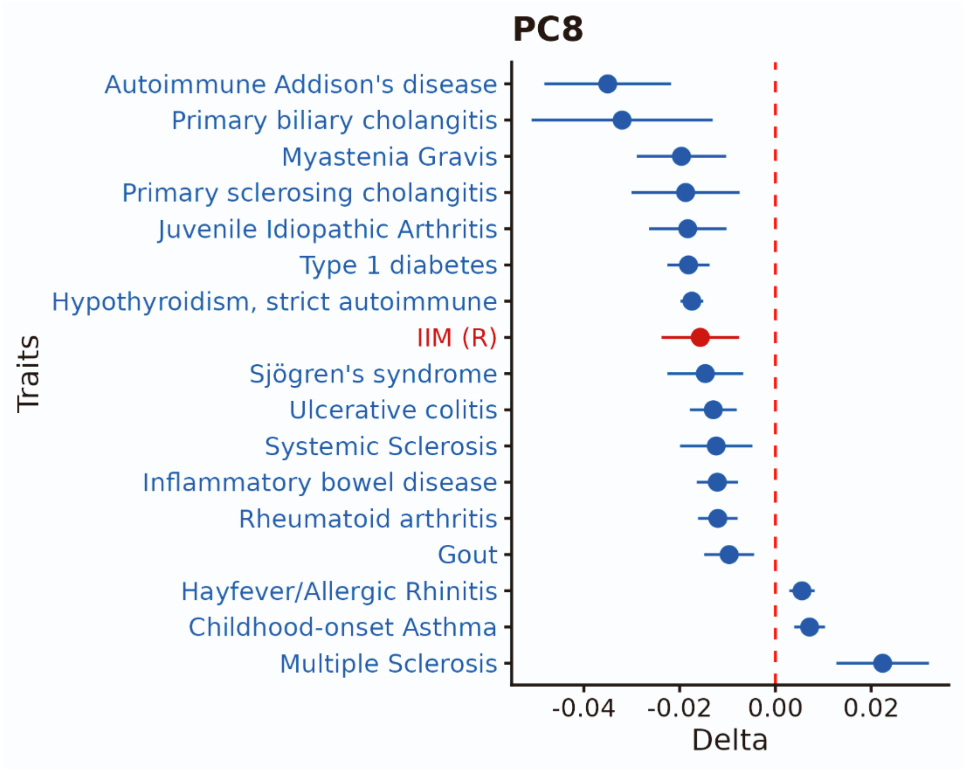

Figure S9 | Delta scores of selected feature-significant IMD at FDR 1% on PC9. Significant myositis datasets are shown in red and IMD in blue.

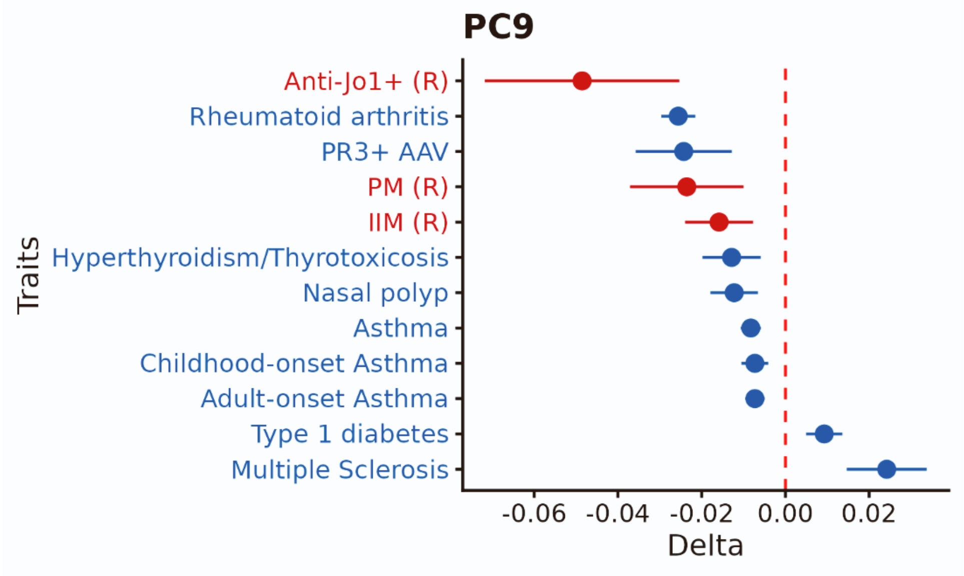

Figure S10 | Delta scores of selected feature-significant IMD at FDR 1% on PC12. Significant myositis datasets are shown in red and IMD in blue.

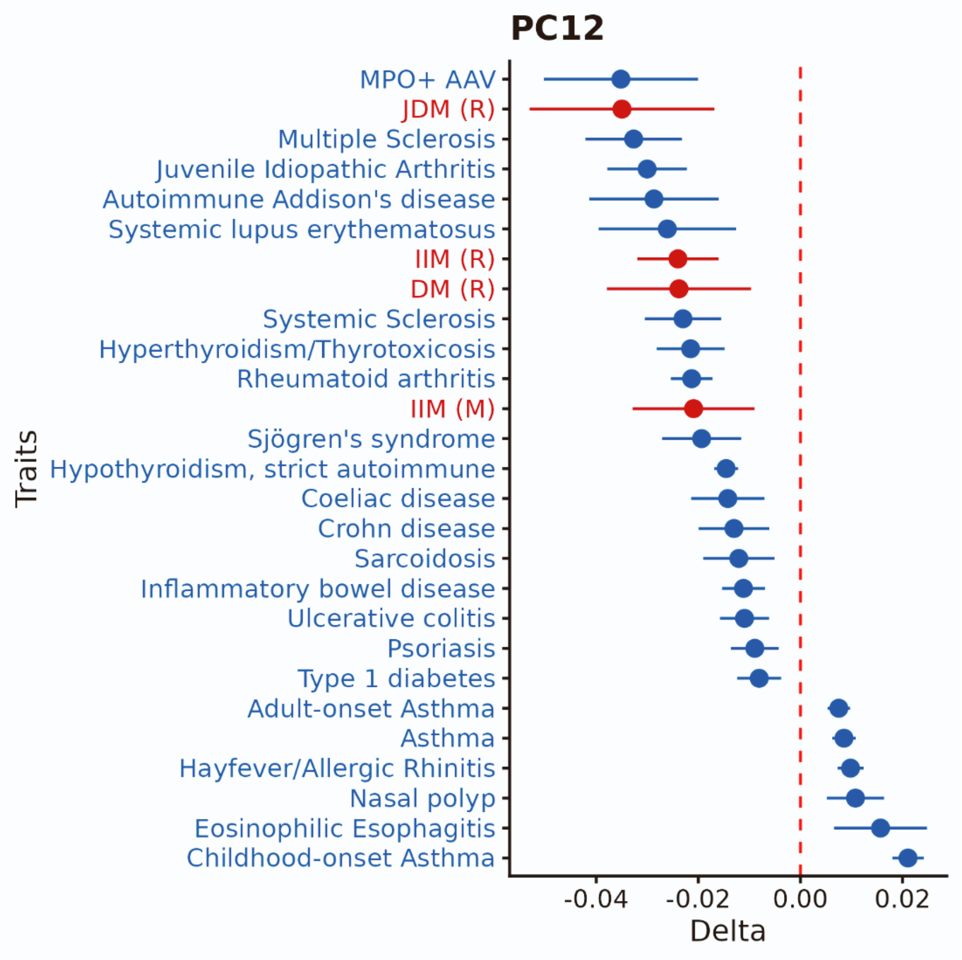

Figure S11 | Delta scores of selected feature-significant IMD at FDR 1% on PC13. Significant myositis datasets are shown in red and IMD in blue.

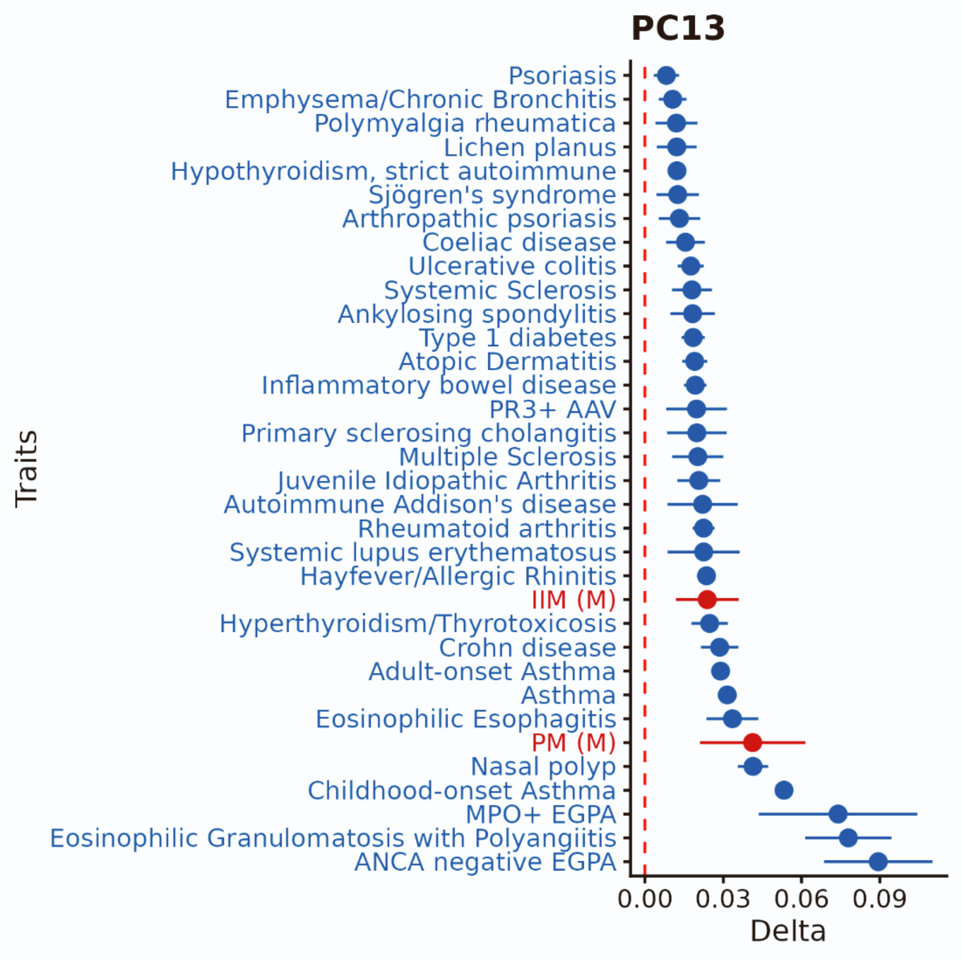

Figure S12 | Hierarchical clustering heatmap on Bhattacharyya distance (DB) between myositis projections and other IMD. Clustering corresponds to DB, while color scale represents log(1 + DB) to facilitate visualization. Clustering annotations on the sides represent the clustering by DB (k = 15) and DPMUnc (k = 10). Myositis traits are shown in bold. A cluster of myositis and close IMD is highlighted by a red rectangle.

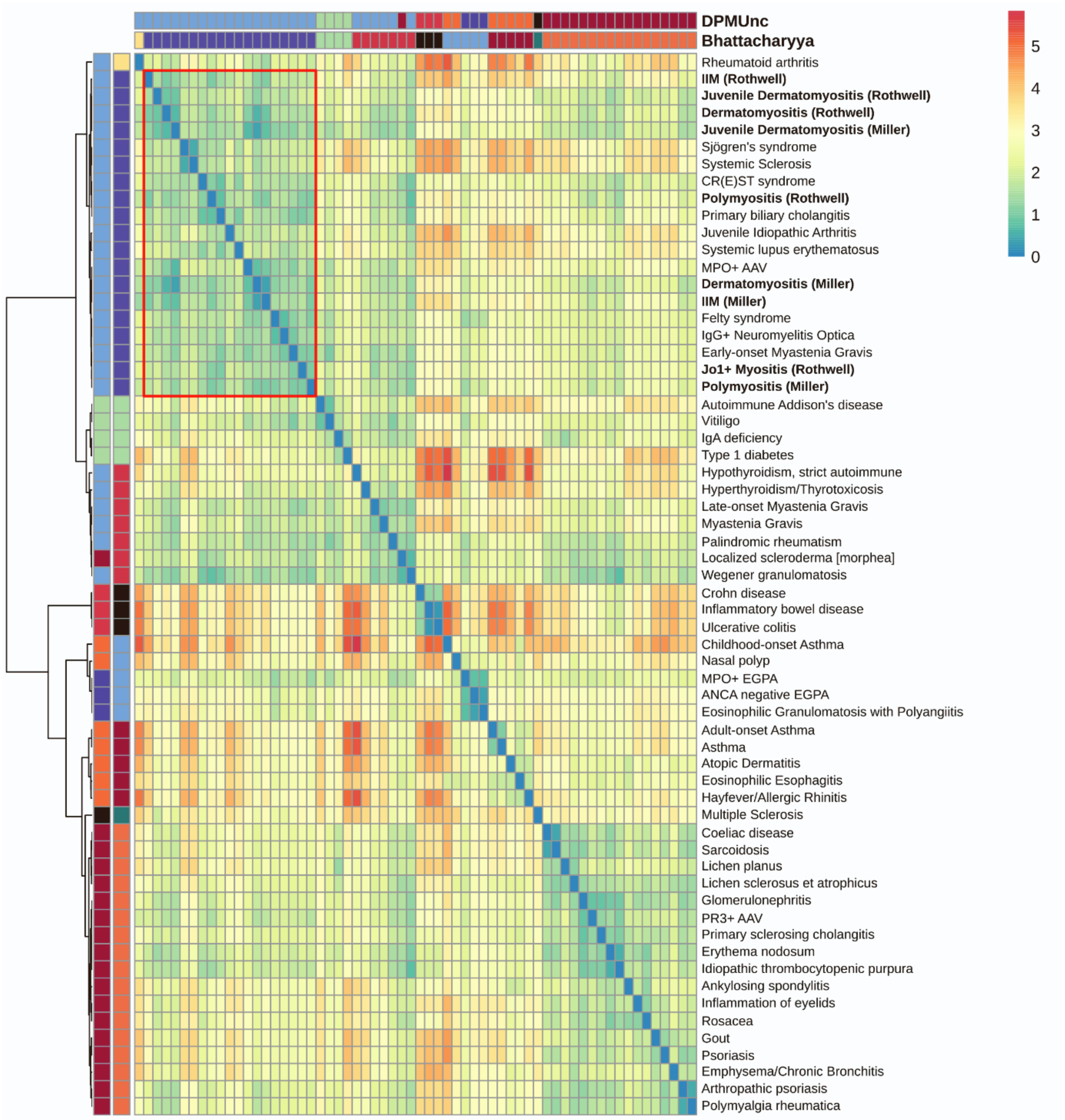

Figure S13 | Pairwise similarity matrix (PSM) based on the posterior probability assigned by DPMUnc for traits to belong to the same cluster. Clustering annotations on the sides represent the clustering by DPMUnc (k = 10) and DB (k = 15). Myositis traits are highlighted in bold.

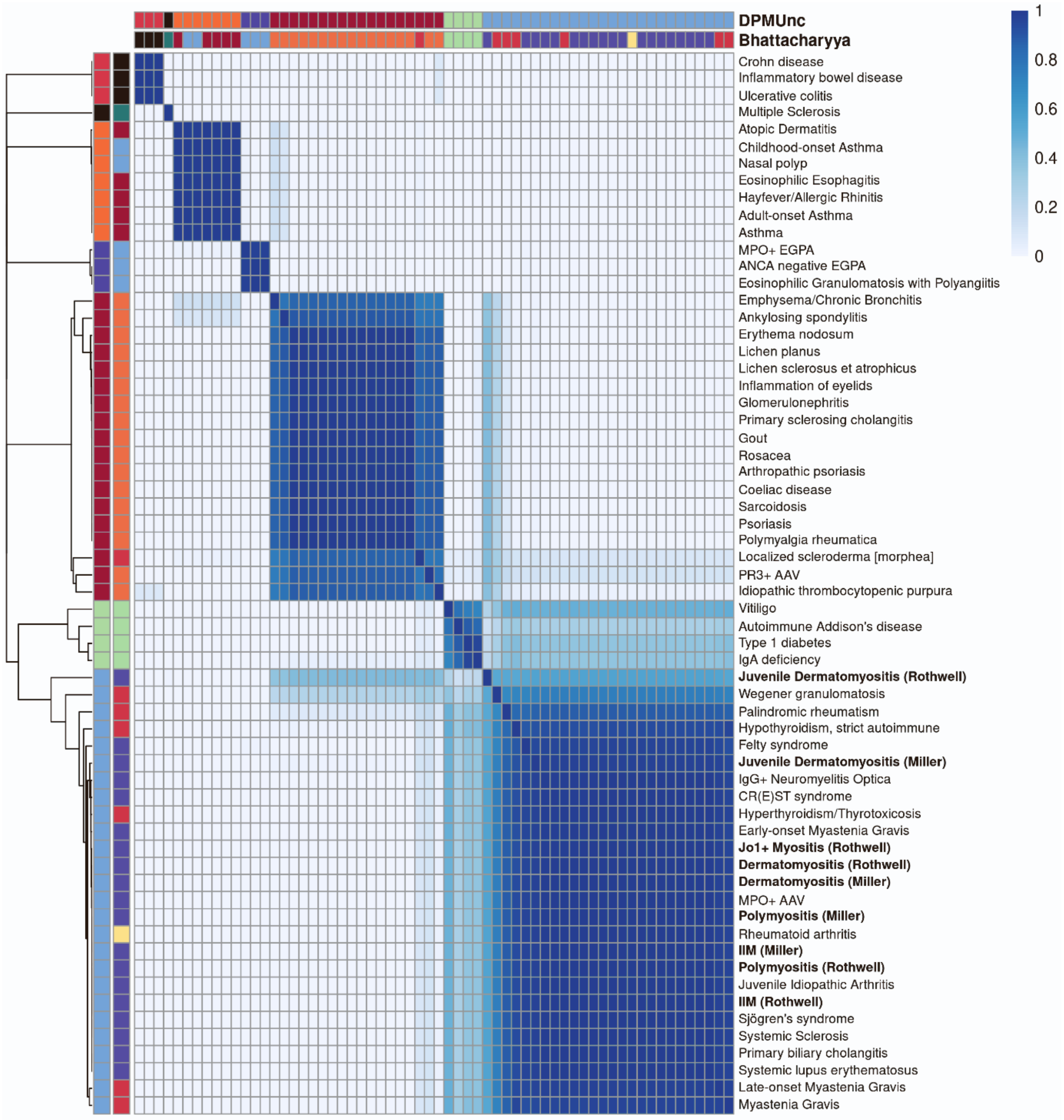

Figure S14 | Effect sizes (Beta) of rs2476601 in seven myositis datasets (excluding IIM meta-analyses). Confidence intervals represent Beta ± SE.

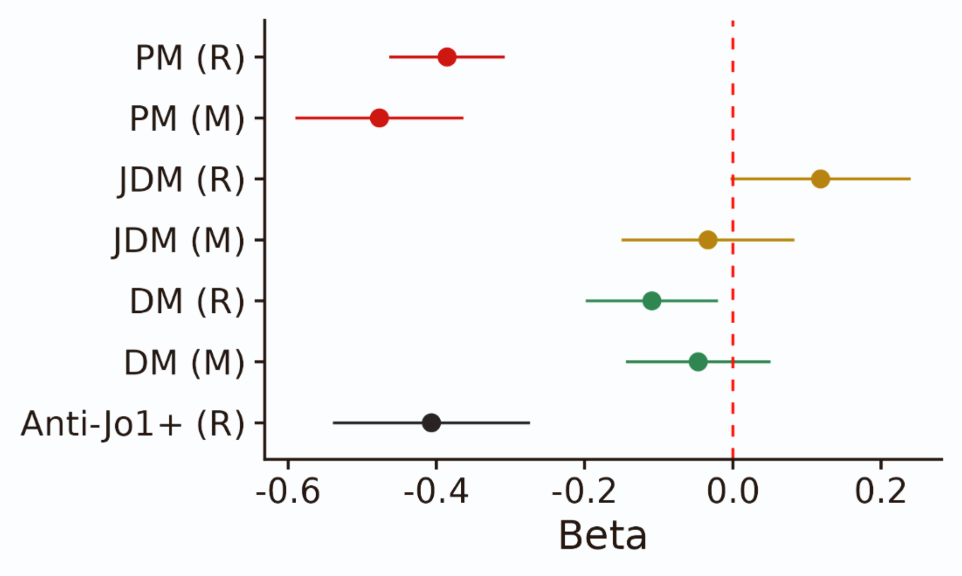

Figure S15 | Delta values of all myositis projections across 7 key features, with 95% CIs.

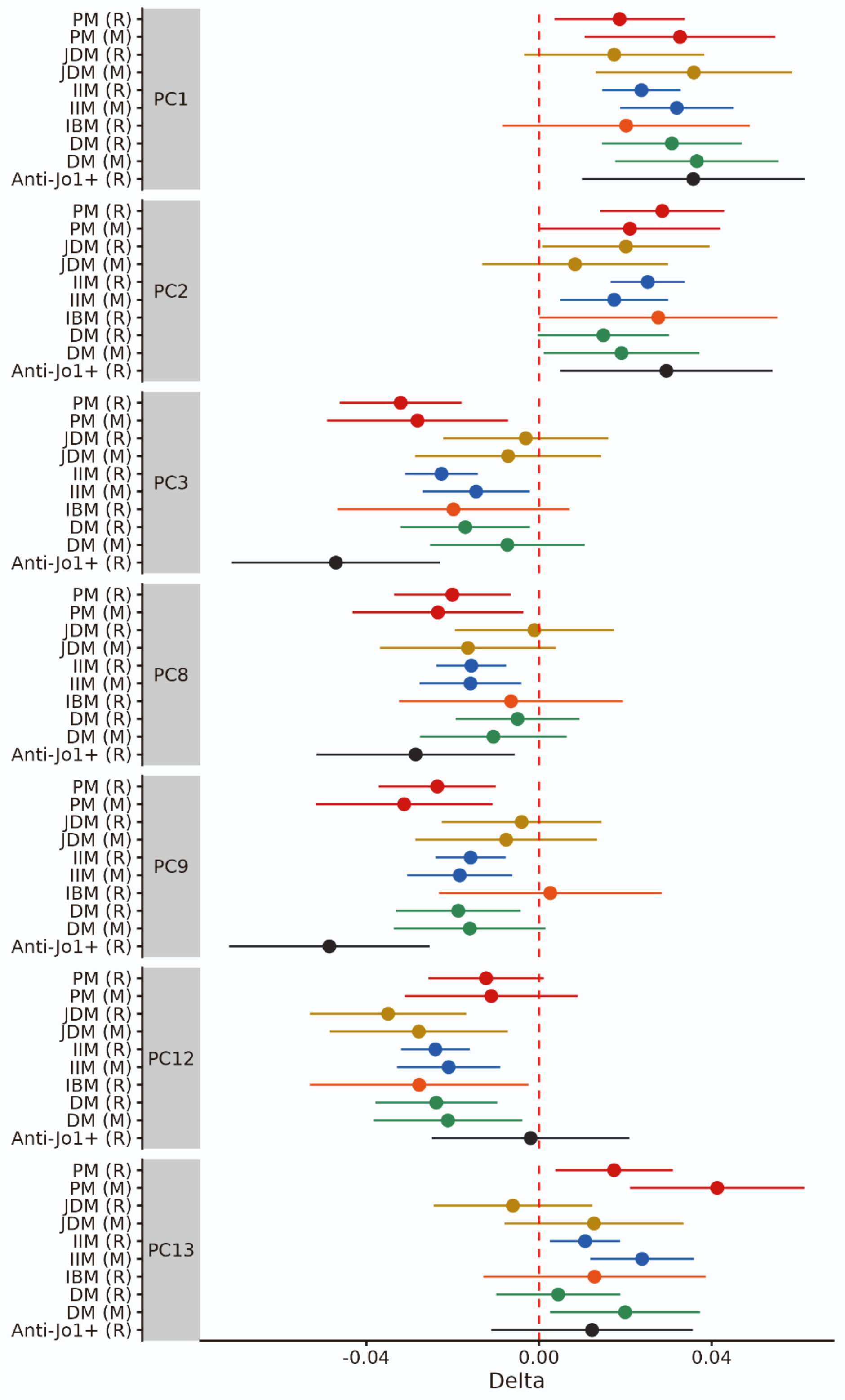

Supplementary Notes

Note S1 - A metric related to the frequency of false positives in a genetic association study with second round data available

We simulated case-control genetic association data at a single SNP as follows. We set the SNP minor allele frequency as

- f = 0.25 in controls
- f = 0.25 in cases (null scenario)
- f = 0.27 in cases (low power scenario)
- f = 0.30 in cases (high power scenario)

and simulated genotypes in 500 cases and 20,000 controls as Binomial(2, f). We tested the association using a t-test. We then simulated a second dataset of the same size, appended it to the first, and repeated the t-test. This gave us two p-values, under round 1 (p1) and round 2 (p2). We repeated these simulations 1,000 times for the high power scenario, 2,000 for the low power scenario and 10,000 for the null scenario (numbers chosen to give a minimum of 500 "significant" tests, p1 < 0.05, in round 1). We compared the proportion of simulations in which p2 < p1 between the given scenarios, *m*, and as a function of the number of null scenarios (false positives) in mixtures across scenarios.

We saw that *m* was close to 0.5 in the null scenarios, and increased with increasing power in the truly associated scenarios, with *m* = 50%, 76%, and 97% for null, low power and high power scenarios, respectively (Figure S3A). However, the difference between scenarios became more pronounced when we conditioned on nominal significance in round 1 (p1 < 0.05), with *m* = 17%, 61%, and 97% for null, low power and high power scenarios, respectively (Figure S3B). In mixtures, *m* increased with decreasing true fraction of false positives, with the strength of relationship depending also on the relative frequency of low and high power scenarios (Figure S3C). We concluded that *m* is proportional to the false positive rate conditional on the combination of power at truly associated variants. As the combination of power is unknown, *m* can be used to compare false positive rates in sets of SNPs for which power at truly associated variants is not expected to systematically differ, but cannot be used to quantify the false positive rate directly.
